# Quantized soliton pumping governed by high-dimensional Chern invariants

**DOI:** 10.1093/nsr/nwag007

**Published:** 2026-01-13

**Authors:** Fengxiao Di, Weixuan Zhang, Hao Yuan, Long Qian, Wenhui Cao, Xiaoqi Zhou, Xiangdong Zhang

**Affiliations:** Key Laboratory of Advanced Optoelectronic Quantum Architecture and Measurements of Ministry of Education, Beijing Key Laboratory of Nanophotonics & Ultrafine Optoelectronic Systems, School of Physics, Beijing Institute of Technology, Beijing 100081, China; Key Laboratory of Advanced Optoelectronic Quantum Architecture and Measurements of Ministry of Education, Beijing Key Laboratory of Nanophotonics & Ultrafine Optoelectronic Systems, School of Physics, Beijing Institute of Technology, Beijing 100081, China; Key Laboratory of Advanced Optoelectronic Quantum Architecture and Measurements of Ministry of Education, Beijing Key Laboratory of Nanophotonics & Ultrafine Optoelectronic Systems, School of Physics, Beijing Institute of Technology, Beijing 100081, China; Key Laboratory of Advanced Optoelectronic Quantum Architecture and Measurements of Ministry of Education, Beijing Key Laboratory of Nanophotonics & Ultrafine Optoelectronic Systems, School of Physics, Beijing Institute of Technology, Beijing 100081, China; Key Laboratory of Advanced Optoelectronic Quantum Architecture and Measurements of Ministry of Education, Beijing Key Laboratory of Nanophotonics & Ultrafine Optoelectronic Systems, School of Physics, Beijing Institute of Technology, Beijing 100081, China; Key Laboratory of Advanced Optoelectronic Quantum Architecture and Measurements of Ministry of Education, Beijing Key Laboratory of Nanophotonics & Ultrafine Optoelectronic Systems, School of Physics, Beijing Institute of Technology, Beijing 100081, China

**Keywords:** non-linear topological pumping, high-order Chern numbers, soliton transport, topolectrical circuits

## Abstract

Non-linear topological pumping—a quantized transport phenomenon of solitary waves in parametrically driven systems—represents a critical interface bridging topological physics and non-linear dynamics. While the first Chern number-governed soliton transport has been extensively studied, the fundamental interplay between high-dimensional band topology and soliton pumping remains unexplored. Here, we establish a theoretical framework and experimental demonstration of soliton topological pumping simultaneously governed by first and second Chern numbers in orthogonal dimensions. Through the modulation of non-linear strength, the system exhibits phase transitions spanning integer-quantized soliton pumping, fractional-quantized soliton pumping and soliton trapping states. Furthermore, by engineering linear band structures, we demonstrate anisotropic soliton pumping where quantized transport manifests integer and fractional characteristics along orthogonal spatial axes. Experimentally, we implement non-linear time-modulated topolectrical circuits whose dynamical equations maintain precise isomorphism with theoretical lattice models, enabling the direct observation of integer, fractional and integer/fractional soliton pumping. Our work establishes a scalable experimental platform for investigating advanced non-linear topological phases, which may be broadly applied to systems at the interface between topological matter and non-linear wave physics.

## Teaser

We reveal quantized soliton pumping governed by high-dimensional Chern invariants, and experimentally observe integer, fractional, and anisotropic soliton pumping in time-modulated nonlinear topolectrical circuits.

## INTRODUCTION

Topological pumping, a cornerstone of modern condensed matter physics, enables quantized particle transport through cyclic adiabatic modulation of periodic potentials [1–19]. This paradigm has recently undergone a non-linear renaissance, where solitons (self-sustained wave packets immune to dispersion) emerge as novel carriers of topological quantization [20–31]. Pioneering work has revealed that weakly non-linear solitons adiabatically track single-band Wannier centers in 1D pumping systems, their quantized motion rigidly locked to the first Chern number [20,21,23]. When the non-linearity exceeds a critical threshold, quantized pumping fractionalizes: solitons follow multi-band Wannier centers, and an integer displacement is completed only after multiple pumping cycles. Such fractional pumping has been established theoretically [23], observed experimentally [25] and further explored in subsequent work [28]. Interestingly, recent studies have demonstrated that integer- and fractional-quantized soliton pumping can also emerge in topologically trivial bands [29], where the non-linearity-induced self-consistent potential modifies the topology of the effective Hamiltonian and thus determines the soliton pumping. These breakthroughs ignited intense exploration of non-linear topological pumping, establishing the interplay between topology and non-linearity as an active frontier of current research.

Beyond 1D, a few studies started to discuss topological soliton transport in 2D pumping systems. The considered models were constructed by two decoupled 1D non-linear Thouless pumping systems. As a result, the center of mass (c.m.) displacements along *x*- and${\mathrm{\ }}y$-axes are independently determined by the first Chern numbers defined in two different 2D parameter subspaces, without involving high-dimensional topological characteristics governed by second Chern numbers [21,22]. This raises a fundamental question: can high-dimensional band topology introduce new physics of non-linear soliton pumping?

The emergence of high-order Chern numbers has significantly advanced our understanding of high-dimensional topological phenomena. The first Chern number underlies 2D quantum Hall physics, while the second and third Chern numbers govern 4D and 6D quantum Hall analogs, which can be experimentally accessed via 2D and 3D topological pumping, respectively [32–36]. For instance, pioneering experimental work [32,33] has demonstrated 2D topological pumping whose quantized response is determined by a second Chern number in photonic and ultracold-atom platforms. Moreover, high-dimensional systems can host even more exotic topological phases, including 4D topological insulators and 5D semimetals [37–39]. However, all of the current results concern high-dimensional Chern responses of topological pumping in linear systems, whereas the interplay between high-dimensional Chern topology and non-linear soliton dynamics has remained largely unexplored. Fundamental questions persist: can non-linearity hybridize quantized transport across dimensions, does strong non-linearity induce fractional pumping, even in high-dimensional settings, and how might distinct Chern numbers of different orders conspire to create novel transport phenomena? Addressing these challenges demands both theoretical frameworks transcending conventional linear topology and experimental platforms capable of probing high-dimensional non-linear dynamics.

Motivated by these considerations, we investigate the interplay between high-dimensional band topology encoded by high-order Chern numbers and non-linear dynamics. Specifically, we resolve these questions through a synergistic theory–experiment approach. By generalizing non-linear pumping to high-dimensional systems governed by distinct-order Chern numbers, we predict and demonstrate dimension-selective quantization—a paradigm where solitons exhibit integer displacement along one spatial axis while undergoing fractional pumping in orthogonal dimensions, dictated by the coordinated action of distinct-order Chern numbers. Recent advances have established electrical circuits as a versatile and highly tunable testbed for non-linear dynamics, with demonstrations including circuit solitons [40] and Lorenz-type chaotic oscillations [41,42], among others. Building on these developments, we design non-linear time-modulated topolectrical circuits that precisely emulate high-dimensional non-linear lattice models, enabling the first direct observation of high-order Chern number-regulated soliton pumping. Our findings not only uncover a new class of non-linear topological phenomena but also establish a versatile platform for exploring uncharted territories where topology, non-linearity and high dimensionality intersect.

## RESULTS

### 2D soliton pumping governed by high-order Chern invariants

**Figure 1.**
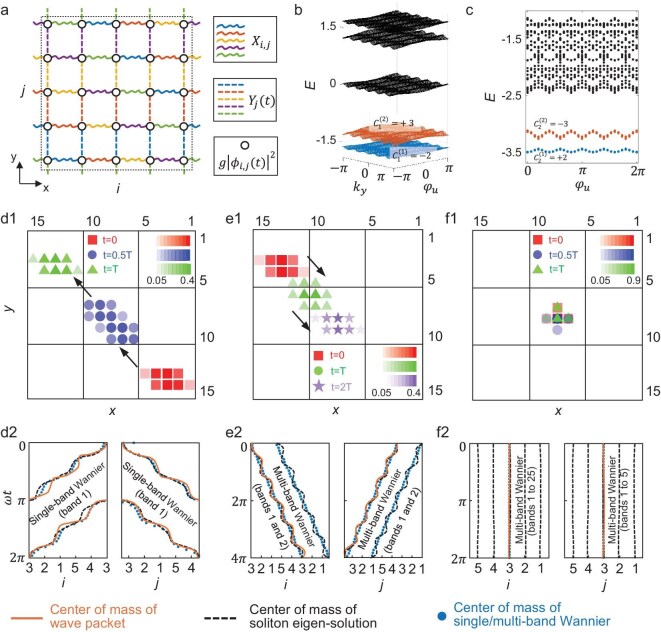
Theoretical results of 2D non-linear Thouless pumping dictated by first and second Chern numbers. (a) Schematic of a 2D Thouless pumping model with 25 sublattices per unit cell, incorporating on-site Kerr non-linearity. The right inset illustrates the intracell coupling terms [${X}_{i,j}$, ${Y}_j( t )$] and the non-linear terms ($g{| {{\phi }_{i,j}} |}^2$). (b) Energy bands of the linear Hamiltonian in the 2D parameter subspace (${k}_y,{\varphi }_u$). The ground state (with Chern number $C_1^{( 1 )} = - 2$) and first excited state ($C_1^{( 2 )} = + 3$) are indicated by blue and red dots, respectively. Higher-energy states are marked with black dots. (c) Low-energy spectra over a full cycle of ${\varphi }_u$ in the full 4D parameter space (${k}_x,{k}_y,{\varphi }_w,{\varphi }_u$), with a full momentum-space scan performed for each ${\varphi }_u$. System parameters are set as ${\varphi }_w = 0.8\pi $, ${J}_x = {J}_y = 0.6$, ${K}_x = {K}_y = 1$, ${\alpha }_{xw} = 0.2$,${\mathrm{\ }}{\alpha }_{yu} = 0.6$, ${B}_{wy} = 0.2{\mathrm{\ and\ }}{\alpha }_0 = 0.8\pi $. (d1–f1) Spatial distributions of the soliton at different times with non-linearity strengths being $g/( {{J}_x + {K}_x} ) = 0.2$, $2.8$, and $5$. Red squares, blue circles, green triangles and purple pentagrams correspond to times: $t = 0$, $t = 0.5T$, $t = T$ and $t = 2T$. Here, *T* is the driving period, and the modulation frequency is $\omega = \partial {\varphi }_u/\partial t = 2\pi /500$. (d2–f2) Center of mass (c.m.) trajectories along *x*- and *y*-axes. Orange solid lines: c.m. trajectories of the soliton [corresponding to (d1–f1)]. Black dashed lines: instantaneous soliton trajectories. Blue dots: c.m. trajectories of single/multi-band Wannier functions.

We start to propose a 2D non-linear Thouless pumping model with the unit cell structure containing 25 sublattices, as enclosed by the black dashed box in Fig. 1a. This model consists of two parts: a linear coupling term and an onsite Kerr non-linearity. The linear component comprises two copies of the Aubry–André–Harper (AAH) model, each residing in an orthogonal 2D subspace labeled as (*x, w*) and (*y, u*). Here, *x* and *y* correspond to spatial dimensions, while *w* and *u* represent synthetic dimensions. Each plaquette in the *xw* (*yu*) plane is pierced by an intrinsic magnetic flux *α_xw_*(*α_yu_*). The nearest-neighbor coupling includes a constant term *J_x_,_y_* and a modulated term *K_x_,_y_*.

To establish a connection between high-dimensional Chern numbers and soliton dynamics, we introduce an external magnetic-field perturbation *B_wy_* in the *wy* plane. Simultaneously, an external electric field *E_u_* along the *u*-axis ensures adiabatic and periodic evolution of the phase *φ*_u_(*t*). Under these conditions, the position-dependent coupling along the *x*-axis at sublattice (*i,j*) is given by ${X}_{i,j} = {J}_x + {K}_xcos( {2\pi [ {{\alpha }_{xw}( {i - 1} ) + {B}_{wy}( {j - 1} )} ] - {\varphi }_w} )$, where $i \in [ {1,{\mathrm{\ }}5} ]$ and $j \in [ {1,{\mathrm{\ }}5} ]$ index the sublattice sites along *x*- and *y*- axes. In contrast, the time- and position-dependent coupling along the *y*-axis is: ${Y}_j( t ) = {J}_y + {K}_ycos( {2\pi [ {{\alpha }_{yu}( {j - 1} )} ] - {\varphi }_u( t ) + {\alpha }_0} )$, where ${\alpha }_0$ is a constant phase. The non-linear contribution $g{| {{\phi }_{i,j}( t )} |}^2$ arises from the onsite Kerr non-linearity, where *g* denotes the non-linear strength and *ϕ_i,j_*(*t*) represents the wavefunction amplitude localized on sublattice (*i,j*) at time *t*. Taking into account both the linear tunnelling and the onsite non-linearity, the dynamics in the 2D lattice are governed by the discrete non-linear Schrödinger equation:


(1) \begin{eqnarray*}
i\frac{\partial }{{\partial t}}{\varphi }_{i,j} (t) &=& {X}_{i,j}{\varphi }_{i + 1,j} (t) + {X}_{i - 1,j}{\varphi }_{i - 1,j} (t)\nonumber\\
&& +\, {Y}_j (t){\varphi }_{i,j + 1} (t) + {Y}_{j - 1} (t){\varphi }_{i,j - 1}(t)\nonumber\\
&& -\, g{\left| {{\varphi }_{i,j} (t)} \right|}^2{\varphi }_{i,j} (t).
\end{eqnarray*}

By slowly varying the pumping parameter *φ_u_*(*t*) in Equation (1) during an adiabatic evolution, we realize a quantized non-linear Thouless pump in the 2D lattice model.

To investigate topological properties of this model, we first compute the band structure in the 2D parameter subspace (*k_y_, φ_u_*) for the linear regime (*g* = 0), corresponding to a 1D AAH model, as shown in Fig. 1b. The ground state, first excited state and higher-energy states are denoted by blue, red and black dots, respectively (see other parameters in Fig. 1 caption). There are two isolated low-energy bands, separated by a large bandgap from other higher-energy bands, satisfying the essential condition for adiabatic evolution. The first Chern number of linear energy bands defined in (*k_y_, φ_u_*) subspace is expressed as $C_1^{yu} = \frac{1}{{2\pi }}\mathop \oint \nolimits_{BZ}^{} \Omega ( {{k}_y,{\varphi }_u} )d{k}_yd{\varphi }_u$, where *Ω*(*k_y_, φ_u_*) denotes the Berry curvature in the (*k_y_, φ_u_*) subspace. Furthermore, we calculate Chern numbers for these two lowest bands, equaling $\{ {C_1^{( 1 )} = - 2,C_1^{( 2 )} = + 3} \}$ with $C_n^{( m )}$ denoting the *n*th Chern number of the *m*th energy band. Extending to the 4D parameter space (*k_x_, k_y_, φ_w_, φ_u_*), we then plot the eigenspectrum of the whole model described by Equation (1) as a function of *φ_u_*, performing full momentum-space scans for each *φ_u_*, as shown in Fig. 1c. Notably, as *φ_u_* varies from 0 to 2*π*, the two lowest bands remain non-intersected and well separated from higher-energy bands, indicating that the topological properties of the system are preserved during the evolution of *φ_u_*. Here, the second Chern number of linear energy bands defined in the (*k_x_, k_y_, φ_w_, φ_u_*) space is expressed as $C_2^{{xwyu}} = \frac{1}{{4{\pi }^2}}\mathop \oint \nolimits_{BZ}^{} \Omega ( {{k}_x,{\varphi }_w} )\Omega ( {{k}_y,{\varphi }_u} )\ d{k}_xd{k}_yd{\varphi }_wd{\varphi }_u$, where $\Omega ( {{k}_x,{\varphi }_w} )$ denotes the Berry curvature in the (*k_x_, φ_w_*) parameter subspace. The second Chern number for two low-energy bands is computed as $\{ {C_2^{( 1 )} = + 2,C_2^{( 2 )} = - 3} \}$, confirming nontrivial high-dimensional Chern numbers.

It is noted that, in the linear regime of the 2D Thouless pumping model, quantized current responses governed by the first and second Chern numbers can emerge along the two spatial directions [32,34]. In this case, the wavefunction is shifted by *C*_2_ and *C*_1_ lattice units along the *x* and *y* directions, respectively, over one pumping cycle. The relationship between the c.m. displacement and the Chern number is derived in Section S1. To verify the quantized transport behavior of our model in the linear limit, we compute the temporal evolution of the wavefunction under the adiabatic evolution of *φ_u_* (see Section S2 for details). Here, although *φ_u_* is the only time-dependent pumping parameter, the presence of *B_wy_* causes ${\varphi ^{\prime}}_w( j ) = 2\pi {B}_{wy}j - \varphi _w^{( 0 )}$ to act as an effective pumping phase along the *x*-direction, resulting in the wave packet shifting along the *x*-direction during the adiabatic cycle. As the initial state, we choose a Wannier state constructed from the lowest band in real space (*x, y*), which carries a trivial first Chern number and is therefore free of topological obstruction.

Due to the band diffraction, the wavefunction spreads radially during the evolution. Nevertheless, the bulk topological properties of the model are clearly revealed by the quantized trajectories of the c.m. along the two spatial directions. Over one pumping period, the c.m. is displaced by $C_2^{( 1 )} = + 2$ and $C_1^{( 1 )} = - 2$ lattice units along the *x*- and *y*-axes, respectively. The c.m. coordinates are defined as:


(2) \begin{eqnarray*}
X_{c.m.} &=& \frac{{\sum\nolimits_i {\sum\nolimits_j {i \cdot {{\left| {{\phi }_{i,j}} \right|}}^2} } }}{{\sum\nolimits_i {\sum\nolimits_j {{{\left| {{\phi }_{i,j}} \right|}}^2} } }},\nonumber\\
Y_{c.m.}&=& \frac{{\sum\nolimits_i {\sum\nolimits_j {j \cdot {{\left| {{\phi }_{i,j}} \right|}}^2} } }}{{\sum\nolimits_i {\sum\nolimits_j {{{\left| {{\phi }_{i,j}} \right|}}^2} } }},
\end{eqnarray*}

where *ϕ_i_,_j_* represents the wavefunction amplitude localized on sublattice (*i,j*).

Building on this linear framework, we next explore how non-linearity modifies the dynamical behavior of 2D topological pumping. Specifically, in the weakly non-linear regime with *g*/(*J_x_* + *K_x_*) = 0.2, we compute the evolution of a soliton initially prepared as a spatially localized instantaneous soliton eigen-solution. It should be emphasized that the instantaneous soliton eigen-solution is a solution of the non-linear eigen-equation. Specifically, at a given time, the non-linear time-evolution equation can be reduced to a non-linear eigen-equation, and the non-linear eigenmode obtained by solving this non-linear eigen-equation using the self-consistency algorithm is precisely the instantaneous soliton at that time (see Section S3 for details).

Red squares, blue circles and green triangles in Fig. 1d1 show the soliton’s spatial distributions at three characteristic times: *t* = 0, *t* = 0.5*T* and *t* = *T*, where *T* is the driving period of *φ_u_*(*t*). Unlike the linear regime, the introduction of non-linearity balances the band diffraction, giving rise to a spatially localized soliton. During the pumping cycle, the soliton exhibits quantized transport behavior. After completing a full period, the soliton returns to its initial state, having been displaced by $C_2^{( 1 )}$ and $C_1^{( 1 )}$ unit cells along the *x*- and *y*-axes, respectively. This demonstrates that weak non-linearity supports integer-quantized soliton pumping along both spatial dimensions, with the transport dictated by the first and second Chern numbers as in the linear regime. In Section S4, we provide a detailed discussion of why the soliton can exhibit quantized Thouless pumping associated with the Chern numbers of the linear bands.

To elucidate the above phenomenon, we further compute the c.m. trajectories of the soliton, projected onto a single unit cell, as shown by orange curves in Fig. 1d2. In this non-linear regime, we find that the soliton ‘tracks’ the instantaneous soliton eigen-solutions (black dashed lines) at every value of time *t* during propagation. Furthermore, we calculate c.m. trajectories of the Wannier functions for the lowest 1D/2D energy bands in Fig. 1b/1c. The c.m. positions of these Wannier states are marked by blue dots. Remarkably, the soliton’s c.m. trajectory faithfully follows the path of these Wannier functions, which exhibit quantization governed by the second Chern number along the *x*-axis and the first Chern number along the *y*-axis. This compelling correspondence demonstrates that the integer-quantized soliton pumping under weak non-linearity is governed by distinct-order Chern numbers along two axes.

As the non-linearity strength increases to *g*/(*J_x_* + *K_x_*) = 2.8, the soliton undergoes a transition from integer-quantized to fractional-quantized pumping along two axes. Figure 1e1 shows the spatial distributions of the soliton at three representative times: *t* = 0, *t* = *T* and *t* = 2*T*, marked by red squares, green triangles and purple pentagrams, respectively. In contrast to the integer-quantized Thouless pumping observed in the weakly non-linear regime, the soliton now propagates in the opposite directions along two axes. After two full driving periods, the soliton’s wavefunction shifts by a unit cell along both spatial directions and returns to its initial profile, indicating a fractional-quantized transport process. This behavior can be understood by analyzing the c.m. trajectories of the maximally localized multi-band Wannier function calculated for the two lowest bands. Notably, in both the 2D parameter subspace (*k_y_, φ_u_*) and the full 4D parameter space (*k_x_, k_y_, φ_w_, φ_u_*), these two lowest bands remain separated by only a narrow bandgap. When the system enters the moderate non-linearity regime, the single-band approximation breaks down, necessitating a multi-band framework to account for inter-band coupling. To address this, we construct maximally localized multi-band Wannier functions from the two lowest bands in each parameter space (see Methods section for construction details). As shown in Fig. 1e2, we observe excellent consistency between the c.m. positions of these multi-band Wannier functions (blue dots) and those of the instantaneous soliton eigen-solution (black dashed lines), with both sets exhibiting two trajectories. The c.m. trajectory of the soliton (orange solid lines) clearly follows one branch of these paths. We emphasize that the alternative trajectory can be realized by choosing a matched initial state. It is worth noting that the c.m. positions of the multi-band Wannier functions along the *x*- and *y*-axes are governed by the averaged topological invariant of two low-energy bands: the averaged second Chern number $C_2^{ave} = ( {C_2^{( 1 )} + C_2^{( 2 )}} )/2 = - 0.5$ for the *x*-axis, and the averaged first Chern number $C_1^{ave} = ( {C_1^{( 1 )} + C_1^{( 2 )}} )/2 = + 0.5$ for the *y*-axis. These findings demonstrate that moderate non-linearity induces fractional-quantized soliton pumping along orthogonal spatial directions, with quantization determined by averaged second and first Chern numbers of two low-energy bands. The theoretical model developed above is implemented on a 5 × 5 lattice of unit cells with periodic boundary conditions. We have verified that the quantized soliton pumping persists for larger system sizes (see Section S5 for details).

It is worth noting that as the non-linearity increases, the evolution from integer-quantized to fractional-quantized soliton pumping does not occur via a sharp phase transition at a single threshold, but instead occurs over a finite crossover regime. Within this regime, the pumping cannot be captured by a simple single-band approximation nor by a straightforward multi-band picture (see Section S6 for details).

Finally, when the non-linearity reaches *g*/(*J_x_* + *K_x_*) = 5, a trapped soliton emerges. As shown in Fig. 1f1, the soliton’s spatial profiles at three representative times, *t* = 0, 0.5*T* and *T*, remain localized around a specific sublattice throughout the entire pumping cycle. This behavior starkly contrasts with integer-quantized and fractional-quantized Thouless pumping, where the soliton propagates across the entire lattice. The underlying mechanism of this strong localization can be understood by analyzing the maximally localized multi-band Wannier functions calculated for all bands combined. Figure 1f2 shows the c.m. trajectories of the soliton (orange solid line), the instantaneous soliton (black dashed lines) and the multi-band Wannier functions (blue dots) along both the *x*- and *y*-axes. All trajectories remain tightly confined near the initial position, indicating a direct correlation between the trapped soliton and the multi-band Wannier functions. Furthermore, the c.m. displacements of the multi-band Wannier functions are governed by the summed Chern numbers: $\mathop \sum \nolimits_{i = 1}^5 C_1^{( i )} = 0$ and $\mathop \sum \nolimits_{i = 1}^{25} C_2^{( i )} = 0$. Specifically, the soliton transport along the *y*-axis is governed by the band topology in the 2D parameter space (*k_y_, φ_u_*). For fixed *φ_u_* the system has only five linear bands, so the relevant first Chern number is obtained by summing over these five bands. In contrast, the transport along the *x*-axis is controlled by the 4D parameter space (*k_x_, k_y_, φ_w_, φ_u_*). Fixing *φ_w_* and *φ_u_* yields 25 bands, and the corresponding second Chern number requires summation over all 25 bands. This implies that the multi-band Wannier functions are highly localized, effectively acting as delta functions centered at individual sublattices. These results confirm that in the strongly non-linear regime, the soliton’s dynamics are influenced by the multi-band Wannier functions of all bands. The vanishing sums of Chern numbers enforce complete spatial confinement of the soliton, leading to its trapped behavior.

The above results show that, by tuning the non-linear strength, the phase transitions between a multiple type of 2D non-linear Thouless pumping appear. In essence, this phenomenon can be understood via non-linear bifurcation, which is associated with spontaneous symmetry breaking and leads to the splitting of the c.m. trajectories for the eigen-solutions of solitons. As the non-linearity increases, the system undergoes two pitchfork bifurcations, corresponding to two distinct transitions in Thouless pumping behavior (see Section S7 for details).

**Figure 2.**
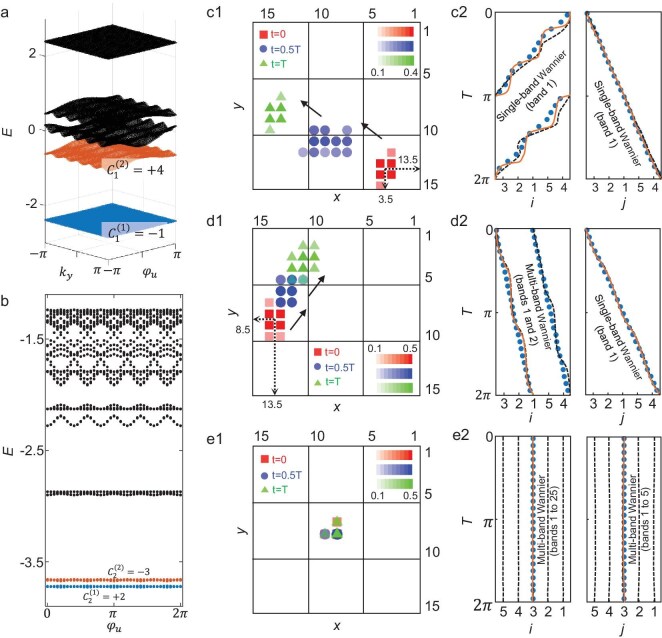
Anisotropic integer/fractional Thouless pumping of solitons along two orthogonal directions. (a) Linear band structure in the 2D parameter subspace (${k}_y,{\varphi }_u$) for intrinsic magnetic fluxes ${\alpha }_{xw} = 0.6$ and${\mathrm{\ }}{\alpha }_{yu} = 0.2$. The ground state and first excited state exhibit nontrivial first Chern numbers $C_1^{( 1 )} = - 1$ and $C_1^{( 2 )} = + 4$, respectively. (b) Numerically calculated low-energy spectrum as a function of ${\varphi }_u$​ over a full cycle in the 4D parameter space (${k}_x,{k}_y,{\varphi }_w,{\varphi }_u$). The system parameters are set to ${\varphi }_w = 0.2\pi $, ${J}_x = 0.61,{J}_y = 0.84$, ${K}_x = {K}_y = 1$, ${B}_{wy} = 0.2{\mathrm{\ and\ }}{\alpha }_0 = 1.6\pi $. (c1, d1, e1) Spatial distributions of the soliton at three characteristic timepoints ($t = 0$, $0.5T$ and *T*) for different non-linearity strengths $g/( {{J}_x + {K}_x} ) = 0.15$, $1.5$ and $6$, respectively. (c2, d2, e2) The c.m. trajectories of the solitons (orange solid lines), the instantaneous soliton eigen-solutions (black dashed lines) and the maximally localized single-band or multi-band Wannier functions (blue dots).

Beyond the synchronous phase transition between integer and fractional soliton pumping along dual axes, in the following, we investigate the anisotropic soliton pumping in two dimensions, that is the soliton exhibits integer- and fractional-quantized pumping along two axes. For this purpose, we set modified values of intrinsic fluxes on *xw* and *yu* planes, *α_xw_* = 0.6 and *α_yu_* = 0.2, respectively. We first compute the linear energy band structure (*g* = 0) as a function of *k_y_* and *φ_u_* in the 2D parameter subspace (*k_y_, φ_u_*), as shown in Fig. 2a. Compared to the results in Fig. 1b, the band structure undergoes significant modifications. Notably, the lowest two bands are now separated by a large bandgap. The associated first Chern numbers change to $\{ {C_1^{( 1 )} = - 1,C_1^{( 2 )} = + 4} \}$. Furthermore, we compute the eigenenergy spectrum in the 4D parameter space (*k_x_, k_y_, φ_w_, φ_u_*) as a function of *φ_u_*, as illustrated in Fig. 2b. Remarkably, similar to Fig. 1c, a finite bandgap persists between the two lowest bands, which remain well separated from higher-energy bands as *φ_u_* varies from 0 to 2*π*. These two bands retain their original nontrivial second Chern numbers, $\{ {C_2^{( 1 )} = + 2,C_2^{( 2 )} = - 3} \}$ compared to those in Fig. 1c. We investigate the Thouless pumping with the modified intrinsic magnetic flux in the linear regime (see Section S8 for details). It is evident that the wavefunction gradually spreads outward during the pumping process. Its c.m. follows an integer-quantized trajectory along both the *x*- and *y*-axes, governed by the second and first Chern numbers, respectively—exhibiting shifts of $C_2^{( 1 )} = + 2$ and $C_1^{( 1 )} = - 1$ lattice units within a single pumping period.

We then explore the Thouless pumping behavior of the system under weak non-linearity *g*/(*J_x_* + *K_x_*) = 0.5. Figure 2c1 shows the spatial distribution of the soliton at three characteristic times: *t* = 0, 0.5*T* and *T*, represented by red squares, blue circles and green triangles, respectively. The initial state corresponds to an instantaneous soliton eigen-solution. In contrast to the integer-quantized soliton pumping behavior observed under the first type of intrinsic magnetic flux—where the transport distances along both spatial directions are identical—here, the soliton exhibits asymmetric displacement: two unit cells along the *x*-axis and one unit cell along the *y*-axis per cycle. After completing one full period, the soliton returns to its initial spatial profile. These results demonstrate that by engineering the intrinsic magnetic flux, one can realize integer-quantized soliton pumping with distinct displacement magnitudes along orthogonal spatial dimensions. To explain this behavior, we calculate the c.m. trajectory of the soliton over one complete pumping cycle along the *x*- and *y*-axes (orange solid curves in Fig. 2c2). Remarkably, the soliton’s c.m. trajectory closely follows that of the maximally localized single-band Wannier functions for the lowest-energy band (blue dots), with quantized displacements determined by the second Chern number $C_2^{( 1 )} = + 2$ along the *x*-direction and the first Chern number $C_1^{( 1 )} = - 1$ along the *y*-direction. Moreover, we compute the c.m. trajectory of the instantaneous soliton eigen-solution (black solid curves), which exhibits excellent consistency with both the soliton and Wannier trajectories.

As the non-linear strength increases to *g*/(*J_x_* + *K_x_*) = 1.5, the soliton’s pumping behavior undergoes a pronounced transformation. Notably, the system exhibits anisotropic integer/fractional 2D soliton pumping: fractional-quantized pumping along the *x*-axis and integer-quantized pumping along the *y*-axis. Specifically, after one full pumping cycle, the soliton shifts by −0.5 unit cell along the *x*-axis and −1 unit cell along the *y*-axis, as illustrated in Fig. 2d1. This phenomenon can be elucidated by analyzing the maximally localized single- and multi-band Wannier functions, as shown in Fig. 2d2. It is found that the soliton’s c.m. trajectory along the *x*-axis closely follows that of the multi-band Wannier functions, which are constructed by combining the two lowest bands in the 4D parameter space. After a full pumping cycle, the c.m. displacement along the *x*-axis corresponds to the average second Chern number $C_2^{ave} = ( {C_2^{( 1 )} + C_2^{( 2 )}} )/2 = - 0.5$. In contrast, the transport along the *y*-axis exhibits a distinct behavior. Due to the large bandgap between the two lowest bands in the 2D parameter subspace (*k_y_, φ_u_*), the inter-band coupling is suppressed at the intermediate non-linearity. Consequently, the *y*-direction motion remains governed by the single-band Wannier function associated with the ground state, yielding a c.m. displacement quantized precisely to the first Chern number of that band. These results demonstrate that, under this modified magnetic flux configuration, increasing non-linearity induces an anisotropic integer/fractional 2D soliton pumping.

At a non-linear strength of $g/( {{J}_x + {K}_x} ) = 6$, the system transitions into a trapped soliton state, as shown in Fig. 2e1, where the soliton remains tightly localized around its initial position throughout the evolution. Figure 2e2 presents the corresponding c.m. trajectories of the soliton, the instantaneous soliton eigen-solution, and the multi-band Wannier functions constructed from all bands. A remarkable consistency among the three trajectories is observed, where the c.m. displacements of the multi-band Wannier functions along both *x*- and *y*-axes vanish, consistent with the vanishing total Chern numbers: $\mathop \sum \nolimits_{i = 1}^{25} C_2^{( i )} = 0$ and $\mathop \sum \nolimits_{i = 1}^5 C_1^{( i )} = 0$.

The above results show that, by engineering linear band structures of the system in 2D and 4D parameter spaces, the anisotropic phase transitions of soliton pumping can be realized along two dimensions. This arises from the interplay between the linear band structures with distinct-order Chern numbers and the non-linear effect. Remarkably, the non-linear strength acts as a control parameter that drives sequential transitions between distinct regimes.

### 2D soliton Thouless pumping in non-linear time-modulated topolectrical circuits

Inspired by recent experimental implementations of topological lattice models using circuit networks [43–56], in this part we theoretically design and experimentally fabricate non-linear time-modulated topolectrical circuits to observe nonlinear 2D Thouless pumping. To enable experimental realization with resistor–capacitor (*RC*) circuits, we transform the lattice model into a purely imaginary-valued version. This modified model comprises time-invariant coupling terms ($\pm i{X}_{i,j}$), time-dependent coupling terms [$\pm i{Y}_j( t )$], and non-linear coupling terms ($\pm ig{| {{\phi }_{i,j}} |}^2$). The corresponding Hamiltonian is given by:


(3) \begin{eqnarray*}
H_{2D}^{mod.} = i\left( {\begin{array}{@{}*{2}{c}@{}} 0&\quad {{H}_{2D}}\\ { - {H}_{2D}}&\quad 0 \end{array}} \right),
\end{eqnarray*}

where ${H}_{2D}$ denotes the Hamiltonian of the original model in Fig. 1a. This modified model retains the same linear eigen-spectrum and non-linearity-induced pumping behaviors as the original model (see Section S9 for details).

**Figure 3.**
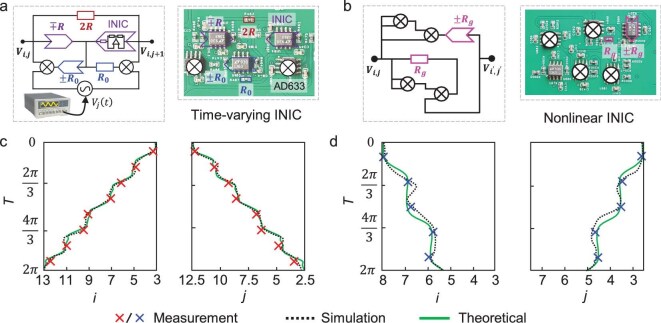
Experimental observation of integer- and fractional-quantized 2D Thouless pumping by non-linear time-modulated topolectrical circuits. (a) Schematic of the time-varying coupling implementation using a time-varying INIC with resistances $R = {R}_0 = 1.5{\mathrm{\ k\Omega }}$. (b) Schematic of the non-linear coupling implementation via a non-linear INIC, where the resistance is ${R}_g = 6.25$  ${\mathrm{k\Omega }}$ in the integer circuit or ${R}_g = 937.5$  ${\mathrm{\Omega }}$ in the fractional circuit. Each circuit node is grounded with a capacitor $C = 100{\mathrm{\ nF}}$. The driving voltages have an amplitude ${V}_0 = 10{\mathrm{\ V}}$ and angular frequency ${\omega }_0 = 11.49{\mathrm{\ Hz}}$ (and ${\omega }_0 = 119{\mathrm{\ Hz}}$ in the fractional circuit). (c and d) Experimentally measured c.m. trajectories of the voltage signal for the integer (red crosses, eight time segments) and fractional (blue crosses, five time segments) circuits, respectively. Black dashed and green solid curves represent the circuit simulations and theoretical results. The pumping periods are $87\ {\mathrm{ms}}$ (integer) and $8.4\ {\mathrm{ms}}$ (fractional).

The time-invariant coupling $\pm i{X}_{i,j}$ between two lattice sites is implemented using a conventional impedance converter with current inversion (INIC) that connects two circuit nodes. The time-dependent coupling $\pm i{Y}_j( t )$ is realized via a time-varying INIC [56], which consists of three conventional INICs, two analog multipliers and two resistors, as illustrated in Fig. 3a. Crucially, by appropriately tuning the external voltage ${V}_j( t ) = {V}_0\,{\mathrm{cos}} [ {{\omega }_0t + \phi ( j )} ]$ injected into one input port of each multiplier, we achieve control over the time- and position-dependent coupling strengths. Specifically, using the circuit design in Fig. 3a, we write Kirchhoff’s current law at the two circuit nodes ($i,j$) and ($i,j + 1$) as:


(4) \begin{eqnarray*}
\left( {\begin{array}{@{}*{1}{c}@{}} {{I}_{i,\!j}}\\ {{I}_{i,\!j + 1}} \end{array}} \right) &=& \frac{{{V}_0\cos[ {{\omega }_0t + \phi ( j )}]}}{{20{R}_0}}\nonumber\\
&&\times \, \left( {\begin{array}{@{}*{2}{c}@{}} 1&\quad{ - 1}\\ 1&\quad{ - 1} \end{array}} \right)
\left( {\begin{array}{@{}*{1}{c}@{}} {{V}_{i,j}}\\ {{V}_{i,j + 1}} \end{array}} \right).
\end{eqnarray*}

In contrast to a conventional INIC, Equation (4) exhibits time- and site-dependent non-reciprocal coupling controlled by the external voltage ${V}_j( t ) = {V}_0 {\rm cos} [ {{\omega }_0t + \phi ( j )} ]$. This time-varying INIC module can be mapped onto the time- and position-dependent coupling term $\pm i{Y}_j( t )$ in the 2D lattice model. In addition, to realize the non-linear coupling $\pm ig{| {{\phi }_{i,j}} |}^2$, we employ a circuit module composed of four multipliers, one INIC and one resistor, as depicted in Fig. 3b. By adjusting the gain coefficients of the multipliers, the input–output relationship of each multiplier is configured as ${V}_{out}( t ) = {V}_{in1}( t ) \times {V}_{in2}( t )$. Applying Kirchhoff’s current law to the two circuit nodes labeled ${V}_{i,j}$ and ${V}_{i^{\prime},j^{\prime}}$ yields:


(5) \begin{eqnarray*}
\left( {\begin{array}{@{}*{1}{c}@{}} {{I}_{i,j}}\\ {{I}_{i^{\prime},j^{\prime}}} \end{array}} \right) = \frac{1}{{{R}_g}}\left( {\begin{array}{@{}*{2}{c}@{}} 1&\quad { - V_{i,j}^2}\\ {V_{i,j}^2}&\quad { - 1} \end{array}} \right)\left( {\begin{array}{@{}*{1}{c}@{}} {{V}_{i,j}}\\
{{V}_{i^{\prime},j^{\prime}}} \end{array}} \right).
\end{eqnarray*}

This configuration realizes a third-order non-linear non-reciprocal coupling between nodes ($i,j$) and ($i^{\prime},j^{\prime}$). A step-by-step circuit derivation of Equations (4) and (5) is provided in Section S10. Finally, each circuit node is grounded with a capacitor. Under this configuration, the voltage dynamical equation at each circuit node ($i,j$) and ($i^{\prime},j^{\prime}$) can be written as:


\begin{eqnarray*}
&&\!\!i\displaystyle\frac{d}{{dt}}{V}_{i,j}\left( t \right) = \frac{i}{{C{R}_{{x}_{i,j}}}}{V}_{i^{\prime} + 1,j^{\prime}} (t ) + \frac{i}{{C{R}_{{x}_{i - 1,j}}}}{V}_{i^{\prime} - 1,j^{\prime}}( t )\nonumber\\
&&\quad + \left( {\frac{i}{{C{R}_{{J}_y}}} + i\frac{{{V}_0\cos [ {{\omega }_0t + \phi ( j)}]}}{{20C{R}_0}}} \right){V}_{i^{\prime},j^{\prime} + 1}( t )\end{eqnarray*}

\begin{eqnarray*}
&&+ \left( {\frac{i}{{C{R}_{{J}_y}}} + i\frac{{{V}_0\cos \left[ {{\omega }_0t + \phi ( {j - 1} )} \right]}}{{20C{R}_0}}} \right)\nonumber\\
&&\quad \times \, {V}_{i^{\prime},j^{\prime} - 1}\left( t \right) - \frac{i}{{C{R}_g}}V_{i,j}^3( t),
\end{eqnarray*}

\begin{eqnarray*}
&&\!\!\! i\frac{d}{{dt}}{V}_{i^{\prime},j^{\prime}}( t) = - \frac{i}{{C{R}_{{x}_{i,j}}}}{V}_{i + 1,j}( t) - \frac{i}{{C{R}_{{x}_{i - 1,j}}}}{V}_{i - 1,j}( t)\nonumber\\
&&\quad - \left( {\frac{i}{{C{R}_{{J}_y}}} + i\frac{{{V}_0\cos [ {{\omega }_0t + \phi ( j)} ]}}{{20C{R}_0}}} \right){V}_{i,j + 1}( t )
\end{eqnarray*}

(6) \begin{eqnarray*}
&&-\, \left( {\frac{i}{{C{R}_{{J}_y}}} + i\frac{{{V}_0\cos [ {{\omega }_0t + \phi ( {j - 1} )} ]}}{{20C{R}_0}}} \right)\nonumber\\
&&\quad \times \, {V}_{i,j - 1}( t ) + \frac{i}{{C{R}_g}}V_{i,j}^3( t),\
\end{eqnarray*}

which take the same form as the modified non-linear time-domain Schrödinger equation at lattice sites ($i,j$) and ($i^{\prime},j^{\prime}$). The voltage signal plays the role of the wave function; the coupling between circuit nodes maps onto the coupling between lattice sites; and the spatial distribution of node-voltage faithfully reflects the soliton profile. The correspondence between the tight-binding model parameters and circuit elements is given by: $\frac{1}{{C{R}_{{x}_{i,j}}}} = {J}_x + \ {K}_x\cos ( {2\pi [ {{\alpha }_{xw}( {i - 1} ) + {B}_{wy}( {j - 1} )} ] - {\varphi }_w} )$, $\frac{1}{{C{R}_{{J}_y}}} = {J}_y$,$\ \frac{1}{{C{R}_g}} = g$, and $\ \frac{{{V}_0\cos [ {{\omega }_0t + \phi ( j )} ]}}{{20C{R}_0}} = {K}_y{\rm cos}( {2\pi [ {{\alpha }_{yu}( {j - 1} )} ] - {\varphi }_u( t ) + {\alpha }_0} )$, with a full derivation provided in Section S11. Within this correspondence, the soliton pumping behavior can be visualized by tracking the spatial distribution of the node-voltage.

At first, we focus on the observation of integer and fractional 2D Thouless pumping of the soliton by fabricating two circuit boards with different non-linear strengths under the first type of intrinsic magnetic flux configuration (${\alpha }_{xw} = 0.2$ and ${\alpha }_{yu} = 0.6$ used in Fig. 1). Due to the strong spatial localization of solitons, the soliton transport behavior can be effectively observed within a single unit cell under a periodic boundary condition (PBC). In this case, each circuit board corresponds to a single unit cell under PBCs. Other circuit parameters are provided in the figure caption. Enlarged photographic images of the time-varying INIC and non-linear INIC are shown in the insets of Fig. 3a and b, respectively. We begin by characterizing integer-quantized Thouless pumping in the non-linear time-modulated topolectrical circuit at a non-linear strength of $g/( {{J}_x + {K}_x} ) = 0.2$. By measuring the voltage signals at all circuit nodes over one complete driving period, we obtain the time-resolved spatial voltage distribution within a single unit cell. To visually present the pumping trajectory in real space, we project this local voltage profile onto the neighboring unit cells that it reaches during the evolution (see Section S12 for further details). We then compute the c.m. coordinates of the voltage profile according to:


(7) \begin{eqnarray*}
{X}_{c.m.} = \frac{{\mathop \sum \nolimits_i \mathop \sum \nolimits_j i \cdot V_{i,j}^2}}{{\mathop \sum \nolimits_i \mathop \sum \nolimits_j V_{i,j}^2}},\quad {Y}_{c.m.}\! = \frac{{\mathop \sum \nolimits_i \mathop \sum \nolimits_j j \cdot V_{i,j}^2}}{{\mathop \sum \nolimits_i \mathop \sum \nolimits_j V_{i,j}^2}}.{\mathrm{\ }}
\end{eqnarray*}

The validity of this projection approach is ensured by the PBCs of the circuit system and the strong localization of the voltage signal.

Furthermore, we extract the c.m. positions of the voltage signal at eight discrete timepoints, marked by red crosses in Fig. 3c. Black dashed and green solid lines represent the circuit simulation (by LTspice) and lattice-theoretical c.m. trajectories along the *x*- and *y*-axes, respectively. It is evident that the experimentally measured c.m. trajectories are matched to circuit simulations and theoretical predictions. Notably, due to parasitic effects and energy dissipation, the experimental results from both the integer and fractional single-unit-cell circuits do not perfectly match the simulations over a full driving period. However, good consistency is observed within the initial portion of the evolution—specifically, within the first $1/8$​ of the driving period for integer circuits and the first $1/5$ for fractional single-unit-cell circuits. To address this, we divide one full driving cycle into several time segments for separate measurements and comparison with the simulation results, which are consistent with the experimental data (see Section S13 for details).

In addition, the topological pumping behavior in our circuit is highly sensitive to resistance disorder, whereas the influence of capacitance disorder on voltage signal transport is comparatively weak. A detailed discussion of the influence of resistive and capacitive disorder on our non-linear topological circuit is provided in Section S14.

We then turn to another circuit sample with the effective non-linear coupling being $g/( {{J}_x + {K}_x} ) = 2.8$ to demonstrate fractional-quantized pumping of the soliton. As shown in Fig. 3d, the experimentally measured c.m. trajectories (blue crosses) along both spatial directions exhibit excellent correspondence with circuit simulations (black dashed lines) and theoretical predictions of fractional-quantized pumping of soliton (green solid lines). Notably, similar to the integer-quantized pumping case, the c.m. evolution is measured over five consecutive time segments.

**Figure 4.**
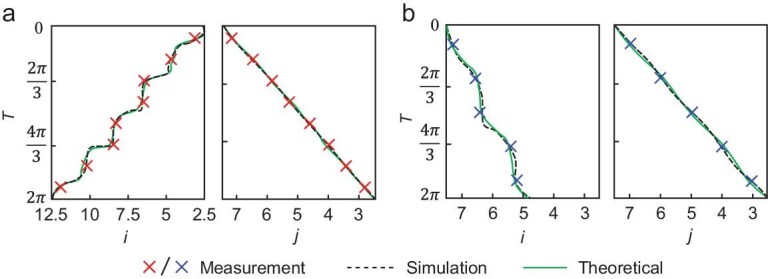
Experimental observation of anisotropic integer/fractional 2D Thouless pumping of solitons by time-modulated topolectrical circuits. (a and b) Experimentally measured c.m. trajectories (red/blue crosses) of the voltage signal in single-unit-cell circuits incorporating non-linear INICs. The non-linear strengths are set to (a) $g/( {{J}_x + {K}_x} ) = 0.15$ (corresponding to ${R}_g = 12.4{\mathrm{\ k\Omega }}$) and (b) $g/( {{J}_x + {K}_x} ) = 1.5$ (corresponding to ${R}_g = 1.24{\mathrm{\ k\Omega }}$). Black dashed and green solid curves represent circuit simulation and theoretical calculation results, respectively. Additional circuit parameters: $R = {R}_0 = 1.5{\mathrm{\ k\Omega }}$, ${V}_0 = 10{\mathrm{\ V}}$ and $C = 100{\mathrm{\ nF}}$. The driving frequencies are (a) ${\omega }_0 = 10.1{\mathrm{\ Hz}}$ and (b) ${\omega }_0 = 119{\mathrm{\ Hz}}$, corresponding to pumping periods of (a) $99\ {\mathrm{ms}}$ and (b) $8.4\ {\mathrm{ms}}$.

Finally, we focus on the observation of anisotropic integer/fractional Thouless pumping of solitons by further designing and fabricating two topolectrical circuits with matched lattice parameters in Fig. 2 (${\alpha }_{xw} = 0.6$ and ${\alpha }_{yu} = 0.2$). Left and right subplots of Fig. 4a show the c.m. trajectories of voltages along the *x*- and *y*-axes, respectively, under a non-linear strength of $g/( {{J}_x + {K}_x} ) = 0.15$ (other circuit parameters are provided in the figure caption). Here, the red crosses, black dashed lines and green solid lines represent the experimental measurements, circuit simulations and theoretical calculations, respectively. We can see that, over a complete driving period, the c.m. of the measured voltage signal shifts by $+ 2$ and $- 1$ lattice units along the *x*- and *y*-axes, respectively, clearly demonstrating integer-quantized pumping with distinct displacement magnitudes along the two spatial directions. Subsequently, we measure the spatial distributions of the voltage signal in another circuit sample with the effective non-linear strength of $g/( {{J}_x + {K}_x} ) = 1.5$. The corresponding c.m. trajectories along the *x*- and *y*-axes are shown in Fig. 4b. It is shown that the experimental results show excellent consistency with circuit simulations and theoretical calculations. Specifically, the c.m. of the voltage signal shifts by $- 0.5$ and$\ - 1$ lattice lengths along the *x*- and *y*-axes, respectively, within one driving period, revealing an anisotropic integer/fractional Thouless pumping of solitons: fractional quantized pumping in the *x*-direction and integer quantized pumping in the *y*-direction.

## DISCUSSION

To further enrich our study, we have also included numerical results on 3D non-linear topological pumping in Section S15. This extension follows a straightforward approach, similar to that demonstrated in linear systems [34,35]. It is shown that quantized integer and fractional soliton transports governed by first, second and third Chern numbers along *z*-, *y*- and *x*-axes can appear. Moreover, by suitably tuning the intrinsic magnetic flux, we further reveal the 3D anisotropic soliton pumping phenomena, such as integer/integer/fractional soliton transport, as supported by theoretical calculations (Section S16) and circuit simulations (Section S17). These results further exhibit the novel interplay between non-linear effects and high-dimensional topological phenomena.

Furthermore, we analyze soliton transport in the presence of a saturable non-linearity. Specifically, we replace the Kerr term $- {g}_s{| \phi |}^2\phi $ with a saturable non-linearity of the form ${f}_{sat}( {{{| \phi |}}^2\phi } ) = - {g}_s\frac{{{{| \phi |}}^2}}{{1 + s{{| \phi |}}^2}}\phi ,$ where *s* is the saturation parameter. We find that, after this replacement, integer, fractional and trapped transport behaviors still persist under appropriate conditions. In this scenario, for fixed non-linear strengths ${g}_s = 0.2( {{J}_x + {K}_x} )$, $2.8( {{J}_x + {K}_x} )$ and $5( {{J}_x + {K}_x} )$, the quantized transport of the soliton can be tuned by varying the parameter *s*. A detailed discussion of soliton transport with a saturable non-linearity is provided in Section S18.

In conclusion, we have theoretically proposed and experimentally demonstrated a novel class of 2D non-linear soliton pumping behaviors governed by distinct-order Chern numbers along different spatial dimensions. By introducing carefully engineered electromagnetic fields in a 4D parameter space, combined with non-linear on-site potentials at each sublattice, we establish a 2D non-linear Thouless pumping model that exhibits second-order topological characteristics. Unlike its linear counterpart, the interplay between non-linear dynamics and high-dimensional Chern invariants gives rise to quantized soliton transport behaviors controlled by distinct-order Chern numbers, including: (i) integer/integer Thouless pumping; (ii) fractional/fractional Thouless pumping; and (iii) integer/fractional Thouless pumping. These emergent behaviors can be understood in terms of Wannier functions displaced by a system-specific topological invariant—the Chern number of the corresponding energy band. Furthermore, we elucidate the physical nature of the non-linearity-induced topological phase transitions by analyzing bifurcation diagrams of the instantaneous soliton c.m. trajectories. To validate our theoretical framework, we have designed and fabricated non-linear time-modulated topolectrical circuits, enabling direct observation of high-dimensional non-linear Thouless pumping governed by distinct-order Chern numbers.

Our work establishes a foundation for exploring high-dimensional non-linear Thouless pumping governed by different orders of Chern numbers. Conceptually, it provides a unified framework that links high-dimensional band topology to the quantized transport of solitons, going beyond both previously studied 1D non-linear and high-dimensional linear Thouless pumping.

On the methodological side, our non-linear time-modulated topolectrical circuits constitute a highly programmable platform in which both the Chern structure and the effective non-linearity can be tuned within a single system. This opens the door to further exploring richer soliton transport behaviors induced by the interplay between non-linear and topological effects. From an application perspective, the demonstrated amplitude-programmable topological soliton pumping suggests practical routes toward topological signal routing, reconfigurable delay lines and amplitude-controlled information processing in topolectrical circuits.

## METHODS

### Construction of maximally localized single/multi-band Wannier functions

As mentioned in the main text, the transport trajectory of solitons follows the centers of maximally localized single-/multi-band Wannier functions, exhibiting integer/fractional pumping behavior. We emphasize that, whether for integer, fractional or trapped soliton transport, the corresponding single-band and multi-band Wannier functions are all defined in the real (*x, y*) space, which has a trivial first Chern number, without being affected by topological obstructions. Below, we introduce the construction methods for single-band and multi-band Wannier functions.

The single-band Wannier state is defined as: $| { {{\omega }_{n,R}} \rangle } = \frac{1}{{\sqrt N }}\mathop \sum \nolimits_k {e}^{ - ikR}| { {{\psi }_{n,k}} \rangle } $, where $| { {{\psi }_{n,k}} \rangle } $ denotes the Bloch state of the *n*th band at wavevector *k, N* is the number of unit cells, and *R* is a lattice vector. These Wannier functions are constructed by uniformly superposing Bloch states across the relevant band and form an orthonormal, complete basis. The localization of single-band Wannier functions depends on the phase choice. To achieve maximal localization, we employ the Marzari–Vanderbilt method. First, we compute the overlap matrix of Bloch states: $M_{mn}^{( {k,b} )} = \\lesssimngle {{{u}_{m,k}}} | {{{u}_{n,k + b}}} \rangle $. A smooth gauge is selected to ensure the Bloch functions remain continuous and differentiable across the Brillouin zone. Next, the overlap matrix $M_{mn}^{( {k,b} )}$ is diagonalized using a unitary matrix $U_{mn}^{( k )}$, such that the phase variation of ${\tilde{M}}^{( {k,b} )} = {U}^{{{( k )}}^\dagger }{M}^{( {k,b} )}{U}^{( {k + b} )}$ is minimized. Upon optimization, ${\tilde{M}}^{( {k,b} )} \approx 1$, ensuring smooth Bloch function phases and highly localized Wannier functions. Finally, the original Bloch states are transformed into new superpositions via ${U}^{( k )}$: $| { {{{\tilde{\psi }}}_{n,k}} \rangle } = \mathop \sum \nolimits_m U_{mn}^{( k )}| { {{\psi }_{n,k}} \rangle } $, yielding the maximally localized Wannier function: $| { {{\omega }_{n,R}} \rangle } = \frac{1}{{\sqrt N }}\mathop \sum \nolimits_k {e}^{ - ikR}( {\mathop \sum \nolimits_m U_{mn}^{( k )}| { {{\psi }_{n,k}} \rangle } } )$.

For multi-band Wannier functions, the definition is: $| { {{\omega }_{\tilde{\beta },R}} \rangle } = \frac{1}{{\sqrt N }}\mathop \sum \nolimits_k {e}^{ - ikR}( {\mathop \sum \nolimits_{m = 1}^{{N}_{{band}}} U_{mn}^{( k )}| { {{\psi }_{m,k}} \rangle } } ),$ where $\tilde{\beta }$ is the Wannier function index ($\tilde{\beta } = 1,2,\ldots,{\mathrm{\ }}{N}_{{band}}$) and ${N}_{{band}}$ is the number of selected bands. Here, the unitary matrix $U_{mn}^{( k )}$ mixes Bloch states from different bands to optimize localization. Unlike single-band Wannier functions, multi-band Wannier functions cannot be associated with a single band but rather with a group of bands, leading to non-uniform occupation across the involved bands. Different gauge choices (i.e. different selections of $U_{mn}^{( k )}$) may result in distinct multi-band Wannier functions and gauge-dependent centers. Only exponentially localized multi-band Wannier functions possess gauge-independent centers. For the 2D case considered in the main text, we require smoothness in all three dimensions (${k}_x,{\mathrm{\ }}{k}_y,t$). Furthermore, we set $U_{mn}^{( k )} = {\delta }_{mn}$, obtaining maximally localized multi-band Wannier functions in the *x*- and *y*-directions. For the 3D case, the Bloch functions must remain smooth across all four spatial dimensions (${k}_x,{\mathrm{\ }}{k}_y,{k}_z,t$). Further details on single- and multi-band Wannier functions can be found in the report by Marzari *et al.* [57].

## Supplementary Material

nwag007_Supplemental_File
