# Supplementary material for: Quantized soliton pumping governed by high-dimensional Chern invariants
Source: Natl Sci Rev. 2026 Jan 13;13(4):nwag007. doi: 10.1093/nsr/nwag007 (PMC12937592; doi:10.1093/nsr/nwag007)
Supplement: nwag007_Supplemental_File [file nwag007_supplemental_file.pdf]

# Supplementary material for

# 'Quantized soliton pumping governed by high-dimensional Chern invariants'

Fengxiao Di[+], Weixuan Zhang[+,*], Hao Yuan, Long Qian, Wenhui Cao, Xiaoqi zhou, and Xiangdong Zhang[*]

*Key Laboratory of advanced optoelectronic quantum architecture and measurements of Ministry of Education, Beijing Key Laboratory of Nanophotonics & Ultrafine Optoelectronic Systems, School of Physics, Beijing Institute of Technology, 100081, Beijing, China*

[+]These authors contributed equally to this work.

[*]Author to whom any correspondence should be addressed: zhangxd@bit.edu.cn; zhangwx@bit.edu.cn

## I. CENTER-OF-MASS DISPLACEMENTS ALONG THE X AND Y DIRECTIONS DETERMINED BY THE SECOND AND FIRST CHERN NUMBERS

In this section, we elucidate the relation between the center of mass (c. m.) displacements along the *x*- (*y*-) directions and the second (first) Chern numbers. Our 2D topological pumping model not only contains two orthogonal 1D AAH models, but also incorporates the effective external EM fields-modulated coupling phases: the *x*-direction coupling $X_{i,j}$ carries a $y$-site–dependent phase $B_{wy}(j-1)$ together with a constant phase $\varphi_w$, while the $y$-direction coupling $Y_j(t)$ includes a time-periodic pumping phase $\varphi_u(t)$ and a constant offset $\alpha_0$. The couplings read:

$$X_{i,j} = J_x + K_x cos\big(2\pi[\alpha_{xw}(i-1) + B_{wy}(j-1)] - \varphi_w\big),$$

$$Y_j(t) = J_y + K_y cos\big(2\pi[\alpha_{yu}(j-1)] - \varphi_u(t) + \alpha_0\big). \quad \text{(S1)}$$

Fig. S1 depicts a single unit cell of the 2D lattice model. It should be emphasized that, for the 2D topological pumping model defined in the $(x, y)$ real space, the initial state must be chosen as a highly localized single-band or multi-band Wannier function defined in the $(x, y)$ space. In this case, after a single pumping period, the displacements along $x$ and $y$ are quantified by the second Chern number (defined in the $(k_x, k_y, \varphi_w, \varphi_u)$ parameter space) and the first Chern number (defined in the $(k_y, \varphi_u)$ parameter space), respectively.

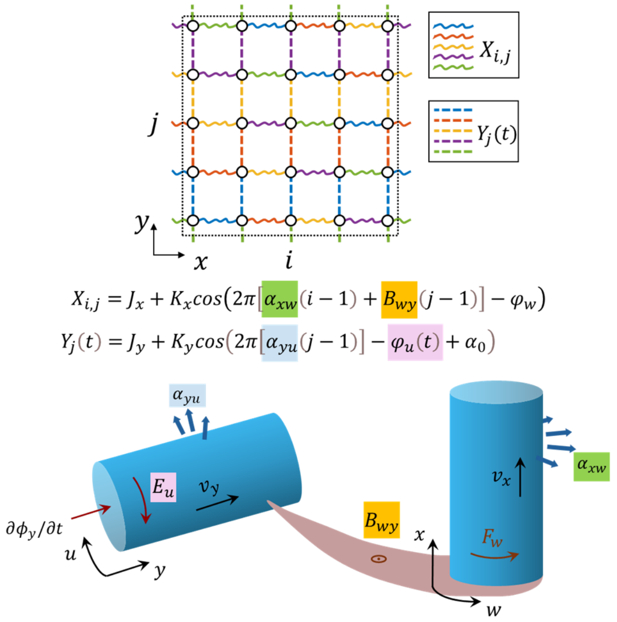


FIG. S1. 2D pumping model defined in real space $(x, y)$ and its corresponding 4D quantum Hall system defined in the parameter space $(k_x, k_y, \varphi_u, \varphi_w)$.

Owing to the $(k_y, \varphi_u)$ parameter space carries a non-zero first Chern number, $C_1^{yu} = \frac{1}{2\pi}\oint_{BZ} \Omega(k_y, \varphi_u)\, dk_y d\varphi_u$, with $\Omega(k_y, \varphi_u)$ the Berry curvature defined in the $(k_y, \varphi_u)$ subspace, the c. m. of the Wannier state defined in $(x, y)$ space acquires a quantized displacement along the $y$ direction. Specifically, the $y$-direction velocity $v_{y,C}$ of the c. m. of the $k_y$ Bloch state can be evaluated from the semiclassical equations of motion [1]:

$$v_{y,C} = \frac{1}{\hbar}\frac{\partial \varepsilon(k_y, \varphi_u)}{\partial t} + \Omega(k_y, \varphi_u)\partial_t \varphi_u, \tag{S2}$$

where $\varepsilon(k_y, \varphi_u)$ is the band energy defined in the $(k_y, \varphi_u)$ subspace. The velocity $v_{y,C}$ has two contributions: the group velocity, arising from the band dispersion, and the anomalous velocity, originating from the nonzero Berry curvature $\Omega(k_y, \varphi_u)$. For a homogeneously populated band in the $(k_y, \varphi_u)$ subspace, the group-velocity term averages to zero for the initial wave packet motion along $y$. In this case, the c. m. displacement (labeled $\Delta Y_C$) is entirely determined by the anomalous term. Consequently, in the 2D pump, when $\varphi_u$ is varied from 0 to $2\pi$, the c. m. shift along $y$ is

$$\Delta Y_C = \frac{1}{2\pi}\int_0^{2\pi}\int_0^{2\pi} v_{y,C} d\, k_y d\varphi_u = C_1^{yu} a_y, \tag{S3}$$

with $a_y$ is the lattice constant.

Importantly, in the presence of $B_{wy}$, the coupling strength along the $x$-axis can be rewritten as

$$X_{i,j} = J_x + K_x cos\big(2\pi[\alpha_{xw}(i-1)] + 2\pi B_{wy}(j-1) - \varphi_w\big)$$

$$= J_x + K_x cos(2\pi[\alpha_{xw}(i-1)] + \varphi'_w(j)), \tag{S4}$$

where $\varphi'_w(j) = 2\pi B_{wy} j - \varphi_w^{(0)}$ acts as an effective pumping phase along the $x$-direction. In this scenario, motion along the $y$-direction during the adiabatic cycle leads to a change in $\varphi'_w$, causing it to undergo effective adiabatic modulation. Neglecting the contribution from the group velocity (which averages to zero for a homogeneously populated band in $k_x$ space), the c. m. velocity of the $k_x$-Bloch wave packet along the $x$-direction becomes

$$v_{x,C} = \Omega(k_x, \varphi_w)\partial_t \varphi'_w = \Omega(k_x, \varphi_w) 2\pi B_{wy} \partial_t j = 2\pi B_{wy} \Omega(k_x, \varphi_w) v_{y,C}$$

$$= 2\pi B_{wy} \Omega(k_x, \varphi_w) \Omega(k_y, \varphi_u) \partial_t \varphi_u. \tag{S5}$$

Here, $\Omega(k_x, \varphi_w)$ is the Berry curvature in the $(k_x, \varphi_w)$ subspace. The c. m. displacement along the $x$-axis (labeled $\Delta X_C$) after a complete cycle can be determined by integrating the velocity over one period. We can thus express the change in the c. m. position per cycle along the $x$-direction as

$$\Delta X_C = \frac{1}{4\pi^2} \oint v_{x,C}\, dk_x dk_y d\varphi_w d\varphi_u = C_2^{xwyu} B_{wy} a_x a_y, \tag{S6}$$

where the second Chern number $C_2^{xwyu}$ is calculated by integrating $\Omega(k_x, \varphi_w)\Omega(k_y, \varphi_u)$ over the entire 4D generalized Brillouin zone

$$C_2^{xwyu} = \frac{1}{4\pi^2} \oint_{BZ} \Omega(k_x, \varphi_w)\Omega(k_y, \varphi_u)\; dk_x dk_y d\varphi_w d\varphi_u. \tag{S7}$$

These results show that, due to the presence of the external coupling magnetic perturbation $B_{wy}$, the change in the electric field $E_u$ in the 4D parameter space during the adiabatic evolution leads to a quantized transport along both the $x$- and $y$-directions, governed by the second and first Chern numbers, respectively. Therefore, in our system, although $\varphi_u(t)$ is the only explicitly time-dependent pumping parameter, the presence of $B_{wy}$ causes the effective phase $\varphi'_w(j)$ to be adiabatically modulated during the evolution, resulting in a shift of the wave packet along the $x$-direction.

## II. NUMERICAL CALCULATIONS OF THE WAVEFUNCTION'S SPATIAL DISTRIBUTIONS AND THE ASSOCIATED CENTER OF MASS DISPLACMENTS IN BOTH THE LINEAR LIMIT AND WEAKLY NONLINEAR REGIMES.

In this section, we numerically investigate the 2D nonlinear Thouless pumping model by analyzing the wavefunction's spatial distributions and c. m. trajectories over one driving period, comparing the linear and weakly nonlinear regimes. In the linear regime (Fig. S2a), we compute the wavefunction's spatial profiles at six equally spaced time points $t = 0$, $0.2T$, $0.4T$, $0.6T$, $0.8T$, and $T$, where $T$

denotes the driving period of $\varphi_u(t)$. The system comprises $5 \times 5$ unit cells with parameters: $\varphi_w = 0.8\pi$, $\varphi_u(t) = 0.8\pi$, $J_x = J_y = 0.6$, $K_x = K_y = 1$, $\alpha_{xw} = 0.2$, $\alpha_{yu} = 0.6$, $B_{wy} = 0.2$ and $\alpha_0 = 0.8\pi$. The initial state is a lowest-band Wannier state constructed from a uniform superposition of all Bloch states within the band. Due to band dispersion, the wavefunction exhibits radial spreading during time evolution. To confirm the quantized current response governed by the first and second Chern numbers, we compute the c. m. trajectory over one driving period and project it onto a unit cell (Fig. S2b). The c. m. displacements are quantized along both spatial directions, with $C_2^{(1)} = +2$ lattice units along the $x$-axis and $C_1^{(1)} = -2$ lattice units along the $y$-axis. These results verify integer-quantized Thouless pumping in the linear regime.

When the nonlinear strength is increased to $g/(J_x + K_x) = 0.2$, the wavefunction's spatial distributions (Fig. S2c) remain localized throughout the evolution, forming stable soliton eigenstates. This behavior stands in stark contrast to the diffractive spreading observed in the linear regime. The c. m. trajectory of the soliton (Fig. S2d) exhibits the same quantized displacements as in the linear case, with $C_2^{(1)} = +2$ along the $x$-direction and $C_1^{(1)} = -2$ along the $y$-direction. These results demonstrate that the system exhibits integer-quantized soliton Thouless pumping in the presence of weak nonlinearity.

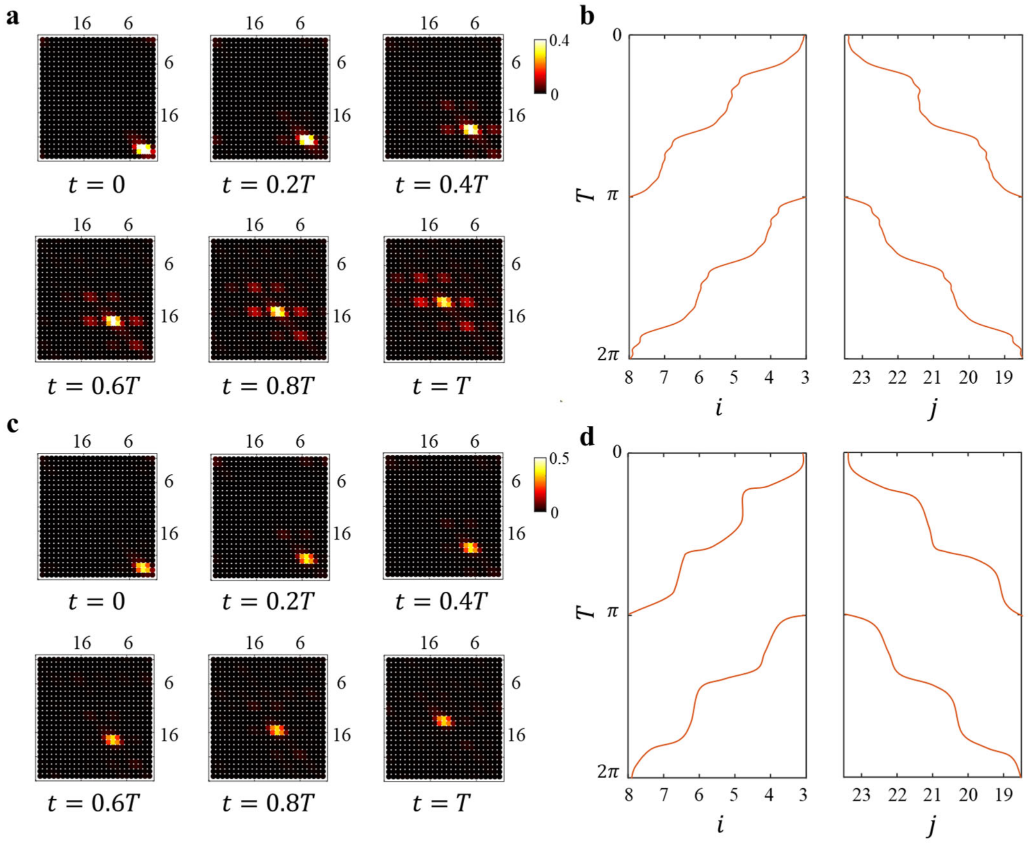


FIG. S2. Numerical results of quantized Thouless pumping in both linear and weakly nonlinear regimes. (a) Wavefunction spatial distributions in the linear limit at six equally spaced time points: $t = 0,\ 0.2T,\ 0.4T,\ 0.6T,\ 0.8T$, and $T$. (b) Corresponding center of mass (c. m.) trajectory over one complete driving period. (c) Numerical results of the soliton's spatial profiles under weak nonlinearity ($g/(J_x + K_x) = 0.2$) at the same temporal sampling points. (d) Associated soliton c. m. trajectory throughout the pumping cycle.

## III. THE DERIVATION OF THE NONLINEAR EIGEN-EQUATION.

In this section, we present the detailed derivation of the nonlinear eigen-equation and the self-consistent algorithm used to solve it.

We start from the discrete nonlinear time-evolution Schrödinger equation (Eq. (1) in the main text):

$$i\frac{\partial}{\partial t}\phi_{i,j}(t) = X_{i,j}\phi_{i+1,j}(t) + X_{i-1,j}\phi_{i-1,j}(t) + Y_j(t)\phi_{i,j+1}(t) + Y_{j-1}(t)\phi_{i,j-1}(t) - g\left|\phi_{i,j}(t)\right|^2\phi_{i,j}(t). \quad \text{(S8)}$$

Writing it in operator form, we obtain

$$i\partial_t\phi(t) = \hat{H}_{lin}(t)\phi(t) - g|\phi(t)|^2\phi(t) \quad \text{(S9)}$$

Here $\hat{H}_{lin}(t)$ is the time-dependent Hamiltonian matrix containing only linear couplings, and $|\phi(t)|^2$ denotes the onsite nonlinear term at each lattice site.

To define the instantaneous soliton, we fix the driving parameter $\varphi_u(\tau)$ at some time $t = \tau$ as

$$Y_j(t) \to Y_j(\tau),$$

At this moment, the driving parameter no longer depends on time, and the Hamiltonian becomes static,

$$\hat{H}_{lin}(t)\big|_{t=\tau} \equiv \hat{H}_{lin}(\tau),$$

Under this static Hamiltonian, we can find a localized, resonant-type time-dependent solution, namely the instantaneous soliton,

$$\phi_{i,j}(t) = \varphi_{i,j}(\tau)e^{-i\varepsilon(\tau)t},$$

where $\varphi_{i,j}(\tau)$ is the spatial wavefunction independent of time (at that moment), and $\varepsilon(\tau)$ is the corresponding "instantaneous nonlinear eigenvalue". Substituting this into Eq. (R8) and simplifying, the left-hand side of the equation becomes

$$i\frac{\partial}{\partial t}\phi_{i,j}(t) = i\frac{\partial}{\partial t}\left[\varphi_{i,j}(\tau)e^{-i\varepsilon(\tau)t}\right] = \varepsilon(\tau)\varphi_{i,j}(\tau)e^{-i\varepsilon(\tau)t}, \quad \text{(S10)}$$

On the right-hand side, the linear-coupling term is

$$X_{i,j}\phi_{i+1,j}(t) + X_{i-1,j}\phi_{i-1,j}(t) + Y_j(t)\phi_{i,j+1}(t) + Y_{j-1}(t)\phi_{i,j-1}(t)$$
$$= \left[X_{i,j}\varphi_{i+1,j}(\tau) + X_{i-1,j}\varphi_{i-1,j}(\tau) + Y_j(\tau)\varphi_{i,j+1}(\tau) + Y_{j-1}(\tau)\varphi_{i,j-1}(\tau)\right]e^{-i\varepsilon(\tau)t}, \quad \text{(S11)}$$

and the nonlinear term is

$$-g\left|\phi_{i,j}(t)\right|^2\phi_{i,j}(t) = -g\left|\varphi_{i,j}(\tau)e^{-i\varepsilon(\tau)t}\right|^2\varphi_{i,j}(\tau)e^{-i\varepsilon(\tau)t} = -g\left|\varphi_{i,j}(\tau)\right|^2\varphi_{i,j}(\tau)e^{-i\varepsilon(\tau)t}, \quad \text{(S12)}$$

Substituting Eqs. (S10), (S11), and (S12) into Eq. (S8) and canceling the overall common factor $e^{-i\varepsilon(\tau)t} \neq 0$, we obtain

$$\varepsilon(\tau)\varphi_{i,j}(\tau) = X_{i,j}\varphi_{i+1,j}(\tau) + X_{i-1,j}\varphi_{i-1,j}(\tau) + Y_j(\tau)\varphi_{i,j+1}(\tau) + Y_{j-1}(\tau)\varphi_{i,j-1}(\tau) - g\left|\varphi_{i,j}(\tau)\right|^2\varphi_{i,j}(\tau), \quad \text{(S13)}$$

Equation (S13) is the nonlinear eigen-equation satisfied by the instantaneous soliton at the fixed instantaneous time $t = \tau$. Its matrix form can be written as

$$\varepsilon(\tau)\varphi(\tau) = \hat{H}_{lin}(\tau)\varphi(\tau) - g|\varphi(\tau)|^2\varphi(\tau). \quad \text{(S14)}$$

Next, we describe the self-consistent algorithm used to solve Eq. (S13).

**Self-Consistent Algorithm**

**1. Initial Trial State:**

We select an initial trial state $\varphi^i$, typically chosen as the Wannier function of the band from which the soliton bifurcates.

**2. Nonlinear Hamiltonian Construction**:

The full nonlinear Hamiltonian $\hat{H}(\tau) = \hat{H}_{lin}(\tau) - g|\varphi^i|^2$ is constructed, where $\hat{H}_{lin}(\tau)$ is the linear

Hamiltonian at time $t = \tau$ and $g$ quantifies the nonlinear strength.

**3. Eigenvector Identification:**

The eigenvector of $\hat{H}(\tau)$ with maximal overlap to $\varphi^i$ is computed and used as the updated trial state for the next iteration.

**4. Convergence Criterion:**

The iteration terminates when the wavefunction satisfies

$$\sum_n \left|\varphi_n^{soliton} - \varphi_n^{ev}\right|^2 \leq 10^{-25}$$

where $\varphi_n^{soliton}$ is the final soliton wavefunction and $\varphi_n^{ev}$ is the eigenvector of $\hat{H}(\tau)$ with the largest overlap.

## IV. A DISCUSSION OF WHY THE SOLITON CAN EXHIBIT QUANTIZED THOULESS PUMPING ASSICIATED WITH THE CHERN NUMBERS OF THE LINEAR BANDS.

In this section, we discuss in detail why a soliton can display quantized nonlinear Thouless pumping associated with the Chern topology of the linear band from two complementary perspectives: (i) the correspondence between the soliton eigenvalues/eigenfields and the linear Bloch bands, and (ii) the correspondence between soliton pumping c. m. trajectories and linear-band Wannier c. m. trajectories.

**(i). Correspondence between the soliton eigenvalues/eigenfields and the linear Bloch bands.** We compute soliton eigenvalues as a function of the nonlinearity strength in the 2D pumping lattice model with $\varphi_u = 0$, as shown in Fig. S3a. Two lowest-energy linear bands are indicated by the grey region. Red and blue solid lines trace the soliton modes bifurcating from these two bands. It is shown that, as the nonlinearity strength increases, solitons bifurcate out of the linear bands and move towards the linear bandgap. In particular, the soliton bifurcating from the second band gradually approaches and eventually enters the lowest band. Moreover, we find that, when $g/(J_x + K_x) = 2.3$, the slope of the lowest-energy soliton eigenvalue as a function of the nonlinearity strength exhibits an abrupt change, indicating that the physical characteristics of the low-energy soliton undergo a qualitative transition. It should be emphasized that different values of the pumping parameter $\varphi_u$ only induce small fluctuations in the nonlinear eigenvalues, while the overall bifurcation structure remains unchanged.

To further analyze the variation of soliton eigenfields, we compute the projection of the soliton wavefunction $\psi_S$ bifurcating from the lowest band onto all Bloch eigenstates $\psi_B$ of the linear bands,

as shown in Fig. S3b. Here we only display the projections onto the eight low-energy bands. Note that, Bloch eigenstates are calculated in the lattice model under periodic boundary conditions with five units along both directions, where 25 Bloch eigenstates are obtained. We find that, in the weak-nonlinearity regime with $g/(J_x + K_x) \in (0.13, 1.7)$, the low-energy soliton almost exclusively occupies the lowest linear band. As the nonlinearity strength increases, its eigenfield projection starts to occupy the Bloch states of the second band ($g/(J_x + K_x) \in (1.7, 2.3)$). Once the nonlinearity strength exceeds the point where the eigenvalue slope abruptly changes, the eigenfield projection becomes nearly evenly distributed over the Bloch states of the lowest two linear bands ($g/(J_x + K_x) \in (2.3, 3.2)$). These results demonstrate that, as the nonlinearity increases, the soliton state bifurcating from the lowest band gradually extends its occupation of the linear Bloch states from the first band to the lowest two bands.

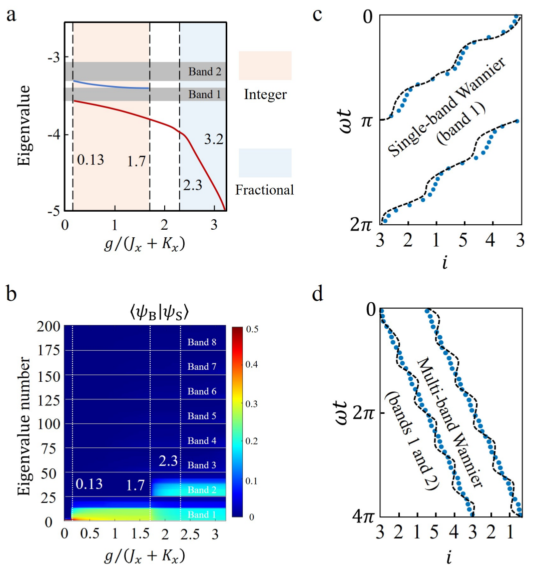


FIG. S3. Numerical results for soliton pumping determined by the Chern numbers of the linear bands. (a) Linear energy bands (grey) for the 2D pumping model at $\varphi_u = 0$ together with the nonlinear eigenvalue of soliton for increasing nonlinearity. The red solid line and blue solid line correspond to solitons bifurcating from the lowest band and the second band, respectively. (b) Projection of the soliton wavefunction onto the linear Bloch eigenstates as a function of the nonlinear strength. The eigenstates are sorted via their eigenvalues from bottom to top. Only the projections onto the lowest eight bands are shown. (c) and (d) Center of mass (c. m.) trajectories of the soliton along the $x$ direction and the corresponding c. m. trajectories of single-band/multi-band Wannier functions in the weak- and moderate-nonlinearity regimes. Black dashed lines: instantaneous soliton. Blue dots: single/multi-band Wannier functions.

(ii). **Correspondence between soliton pumping c. m. trajectories and linear-band Wannier c. m. trajectories.** In linear Thouless pumping, quantized charge transport can be understood in terms of Wannier functions: over one adiabatic cycle, the displacement of a Wannier center is fixed by the topological invariant of the associated band. From the numerical results discussed above, we see that the soliton's occupation of the linear Bloch states changes significantly as the nonlinearity strength is increased. In the following, we demonstrate that this change in Bloch-state occupation leads to a corresponding modification of the relation between the soliton pumping trajectory and that for the Wannier functions.

To verify this, we first focus on the weak-nonlinearity regime (yellow region in Fig. S3(a)). For a nonlinearity strength $g/(J_x + K_x) = 0.2$, we compute the c. m. of the soliton as a function of the pumping parameter $\varphi_u(t) = \omega t$, and obtain its trajectories along the $x$ direction, plotted as black dashed lines in Fig. S3c. The c. m. trajectories of the maximally localized Wannier function constructed from the lowest linear bands are shown as blue dots. Since in the weak-nonlinearity regime, the soliton state fully occupies the first linear band, the soliton's c. m. trajectory faithfully follows the path of the single-band Wannier functions belonging to this linear band. We then increase the nonlinearity strength to $g/(J_x + K_x) = 2.8$ and calculate the soliton c. m. trajectories, shown as the black dashed lines in Fig. S3d. The c. m. trajectories of the maximally localized two-band Wannier functions constructed from the two lowest linear bands are plotted as blue dots. We find excellent consistency between the c. m. positions of these multi-band Wannier functions and those of the soliton, with both sets exhibiting two trajectories. This behavior is fully consistent with the above result that, in this nonlinearity regime, the soliton eigenfield occupies the two lowest linear bands.

Finally, we note that soliton pumping along $y$-axis is determined by linear band topology in $(k_y, \varphi_u)$ subspace. Similar to the above results for pumping along $x$-axis, the corresponding soliton c. m. transport trajectories along $y$-axis are consistent with the Wannier centers defined in 1D pumping model, as already demonstrated in Ref. [2].

## V. NUMERICAL RESULTS OF QUANTIZED SOLITON PUMPING IN LARGER SYSTEM SIZES.

In this section, we present the numerical results of fractional-quantized soliton pumping for larger system size. In addition to the 5 * 5 lattice we further calculated the system's fractional transport for

lattice sizes 7 * 7, 10 * 10, and 13 * 13, as shown in Fig. S4.

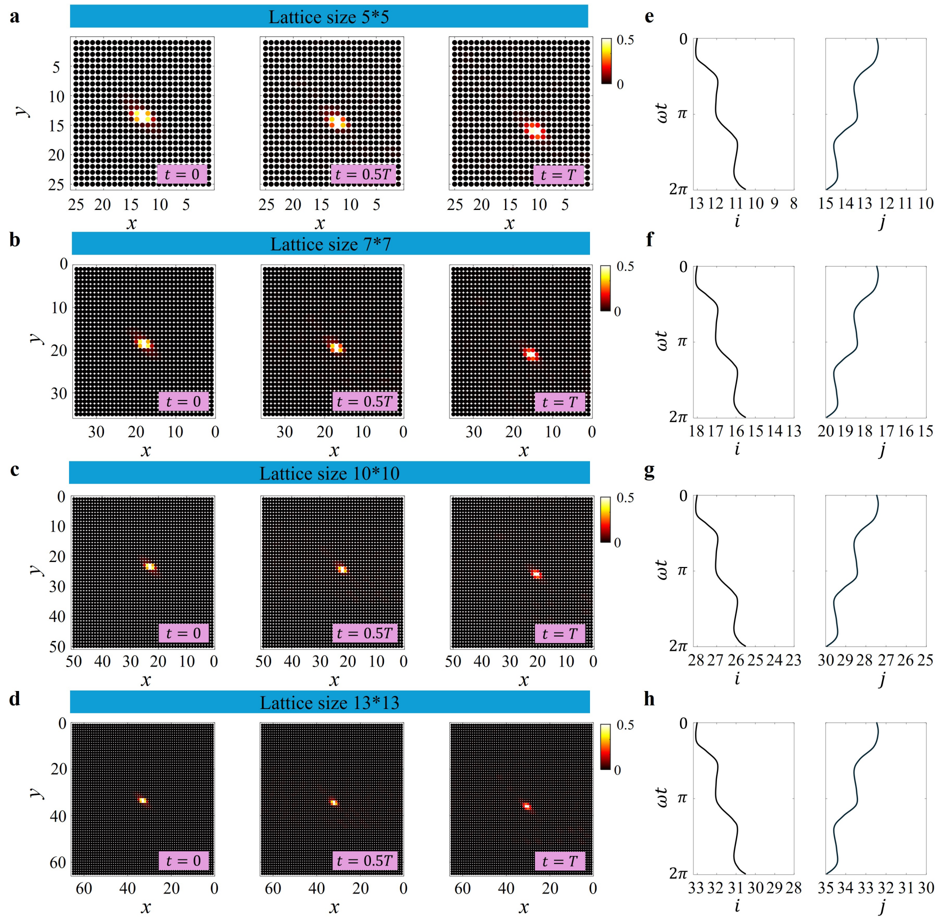


FIG. S4. The fractional-quantized pumping behavior in larger lattice sizes. (a) In the 5 * 5 lattice size, the spatial distribution of the wave packet at three characteristic times: $t = 0$, $t = 0.5T$, and $t = T$, where $T$ is the driving period of $\varphi_u(t)$. The corresponding c. m. transport trajectory over one pumping period is shown in (e). (b)-(d) The spatial distributions for the larger sizes 7 * 7, 10 * 10, and 13 * 13; the corresponding c. m. transport trajectories are shown in (f)-(h), respectively.

Figs. S4a-4d show the soliton spatial profiles at the characteristic times $t = 0$, $t = 0.5T$, and $t = T$ for lattice sizes 5 * 5, 7 * 7, 10 * 10, and 13 * 13 (with $T$ being the driving period of $\varphi_u(t)$). Other parameters are the same as in Figure. 1(e1) of the main text. The corresponding c. m. transport trajectories are shown in Figs. S4e-4h. We can see that, during the adiabatic evolution, the wave-packet spatial distributions and c. m. trajectories at larger sizes are consistent with those for the 5 * 5 case, indicating that fractional-quantized soliton transport persists at larger sizes. For the other (integer and trapped) quantized transport behaviors, the same conclusion holds. They also persist for sufficiently large sizes.

## VI. DETAILED ANALYSIS OF THE PHASE TRANSITION IN SOLITON PUMPING WITH INCREASING NONLINEARITY

In this section, we give a more detailed account of how the soliton pumping evolves with increasing nonlinearity strength, going from integer-quantized to fractional-quantized and, eventually, to a trapped regime.

It is noted that the crossover from integer- to fractional-quantized pumping, as well as the subsequent crossover from fractional-quantized to trapped behavior, is not a sharp phase transition characterized by a single critical value of $g$, but instead occurs over a finite crossover regime. To make this point clear, we first define the average c. m. displacement along the $x$ and $y$ directions as

$$\langle X_C \rangle = \frac{\Delta X_C}{\Delta T}, \quad \langle Y_C \rangle = \frac{\Delta Y_C}{\Delta T},$$

where $\Delta X_C$ and $\Delta Y_C$ denote the c. m. shift along $x$- and $y$-axes over one pumping cycle of duration $\Delta T$. Fig. S5 shows the dependence of $\langle X_C \rangle$ and $\langle Y_C \rangle$ on the nonlinearity strength. For each value of the nonlinearity, the initial state is chosen as the instantaneous soliton eigen-solution at the corresponding nonlinearity strength, and all other parameters are the same as in Fig. 1 in the main text.

In the weakly nonlinear regime (blue shaded region), $\langle X_C \rangle$ and $\langle Y_C \rangle$ in the two directions are locked at $\pm 2$, exhibiting integer-quantized pumping. In this regime, the transport along both directions is governed by the first/second Chern numbers of the lowest band, and the single-band approximation holds simultaneously for $x$- and $y$-axis pumping cases. As $g$ increases, the average c. m. displacements in both directions start to deviate from $\pm 2$ and enter a crossover region (green shaded region). Within this regime, $\langle X_C \rangle$ and $\langle Y_C \rangle$ are neither integer ($\pm 2$) nor on the fractional plateau ($\pm 1/2$). Consequently, this regime cannot be simply attributed to the single-band approximation, nor be regarded as multi-band framework. In other words, within this regime there is no situation in which one direction exhibits an integer plateau while the other has already developed a fractional plateau.

When $g/(J_x + K)$ is further increased beyond a certain threshold, the average c. m. displacements in the two directions undergo a simultaneous jump and settle into the fractional plateau $\langle X_C \rangle = -1/2$ and $\langle Y_C \rangle = +1/2$ (red shaded region). In this regime, the soliton pumping behavior can be well described by a multi-band Wannier function involving the two lowest linear bands: the fractional displacement along $x$ is determined by the second Chern numbers of these two bands, while the

fractional displacement along $y$-axis is determined by their first Chern numbers.

For even larger nonlinearity, a narrow crossover region (purple shaded region) also appears between the fractional plateau and the trapped regime. Beyond this crossover, $\langle X_C \rangle$ and $\langle Y_C \rangle$ both approach zero (yellow shaded region), corresponding to a trapped soliton in the strongly nonlinear regime.

The above nonlinear transition is similar to the numerical results for multi-band fractional pumping in the one-dimensional off-diagonal AAH model [2]: in different nonlinearity regimes, one observes integer and several fractional plateaus, but these plateaus are always separated by crossover regions of finite width, within which simple single-band or multi-band topological descriptions no longer strictly apply.

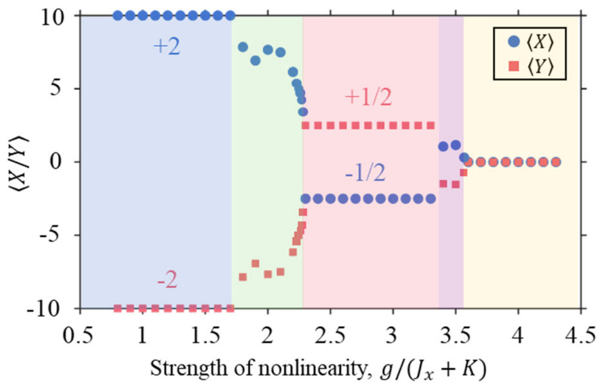


FIG. S5. Average c. m. displacement, $\langle X_C \rangle$ and $\langle Y_C \rangle$, calculated for a 2D nonlinear Thouless pumping model. Blue circles and red squares represent the average c. m. displacements of the soliton along the $x$ and $y$ directions, respectively. System parameters are set to $\varphi_w = 0.8\pi$, $J_x = J_y = 0.6$, $K_x = K_y = 1$, $\alpha_{xw} = 0.2$, $\alpha_{yu} = 0.6$, $B_{wy} = 0.2$ and $\alpha_0 = 0.8\pi$. The blue, green, red, purple, and yellow shaded regions indicate, respectively, the integer-quantized regime, the crossover from integer to fractional pumping, the fractional-quantized regime, the crossover from fractional to trapped pumping, and the trapped regime.

## VII. NONLINEAR BIFURCATION IN THE 2D THOULESS PUMPING MODEL

In this section, we numerically investigate the topological phase transition in the transport behavior of solitons induced by nonlinear bifurcations in a 2D Thouless pumping model. This is achieved by computing the c. m. positions of instantaneous soliton eigen-solutions at four nonlinear strengths: $g/(J_x + K_x) = 0.2,\ 2.8,\ 3.6,$ and $5$. Figs. S6a-S6d show the c. m. trajectories of stable soliton eigen-solutions along the $x$-direction (left panel) and $y$-direction (right panel) over one driving period. The system parameters are identical to those in Section S2, and the initial guess state is chosen as the Wannier

function of the lowest band.

In this 2D Thouless pumping model, the soliton eigen-solutions exhibit mirror-symmetric and continuous paths in both spatial directions. Therefore, we focus on the evolution of the c. m. trajectory evolution along the y-axis. Fig. S6a with $g/(J_x + K_x) = 0.2$ shows two continuous branches, corresponding to displacements of $-2$ and $+3$ unit cells, consistent with the first Chern numbers of the lowest two bands in the 2D subspace $(k_y, \varphi_u)$. As the nonlinearity increases, the system undergoes a pitchfork bifurcation, where trajectories split at their intersection point, generating additional soliton eigen-solutions. At $g/(J_x + K_x) = 2.8$ (Fig. S6b), these split paths reorganize into two new continuous trajectories, each exhibiting a fractional displacement of $+1/2$ unit cells per cycle, indicating a transition from integer-quantized to fractional pumping. This configuration remains robust over a broad range of nonlinearity.

At $g/(J_x + K_x) = 3.6$ (Fig. S6c), a second pitchfork bifurcation occurs, quadrupling the number of soliton eigen-solutions and breaking the continuity of the c. m. trajectories. When the nonlinearity is further increased to $g/(J_x + K_x) = 5$ (Fig. S6d), the number of solutions increases further, and their c. m. positions cluster around the initial sublattice sites, indicating the emergence of trapped solitons. Fig. S6e shows the bifurcation diagram of the soliton eigenstates' c. m. positions as a function of nonlinear strength at $t = 0.3T$.

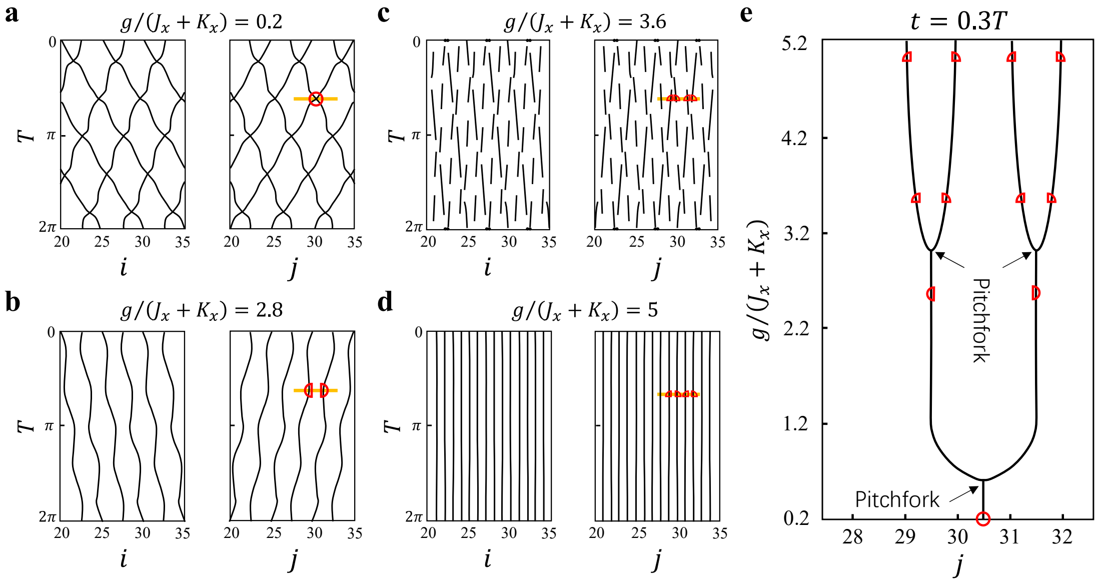


FIG. S6. Mechanism of nonlinear pumping. (a)-(d) C. m. trajectories of soliton eigen-solutions during one pumping cycle along $x$- and $y$-directions (left and right panels, respectively). (e) Bifurcation diagram of nonlinear eigenstates along the $y$-direction at $t = 0.3T$, as a function of nonlinearity strength. Red symbols label specific soliton positions

of different branches, with the yellow solid line indicating one unit cell length, as shown in (a)-(d). The system contains thirteen unit cells per spatial direction with parameters identical to Fig. 2.

## VIII. NUMERICAL RESULTS OF THE 2D THOULESS PUMPING MODEL IN THE LINEAR REGIME UNDER MODIFIED INTRINSIC MAGNETIC FLUXES.

In this section, we numerically investigate the transport behavior of the 2D Thouless pumping model in the linear regime under modified intrinsic magnetic fluxes, specifically $\alpha_{xw} = 0.6$, $\alpha_{yu} = 0.2$. Fig. S7a displays the spatial distributions of the wavefunction at six time points—$t = 0,\ 0.2T,\ 0.4T,\ 0.6T,\ 0.8T$, and $T$—where $T$ denotes the driving period. The initial state is chosen as a Wannier state of the lowest energy band in the 4D parameter space $(k_x, k_y, \varphi_w, \varphi_u)$. It is evident that the wavefunction gradually spreads outward during the pumping process, exhibiting similar transport behavior similar to that in Fig. 1(a).

We further compute the c. m. trajectory of the wavefunction over one full pumping cycle, as illustrated in Fig. S7b. The wavefunction shifts by $C_2^{(1)} = +2$ and $C_1^{(1)} = -1$ lattice constant along the x- and y-axes, respectively, demonstrating integer-quantized Thouless pumping with distinct displacement magnitudes along orthogonal spatial directions.

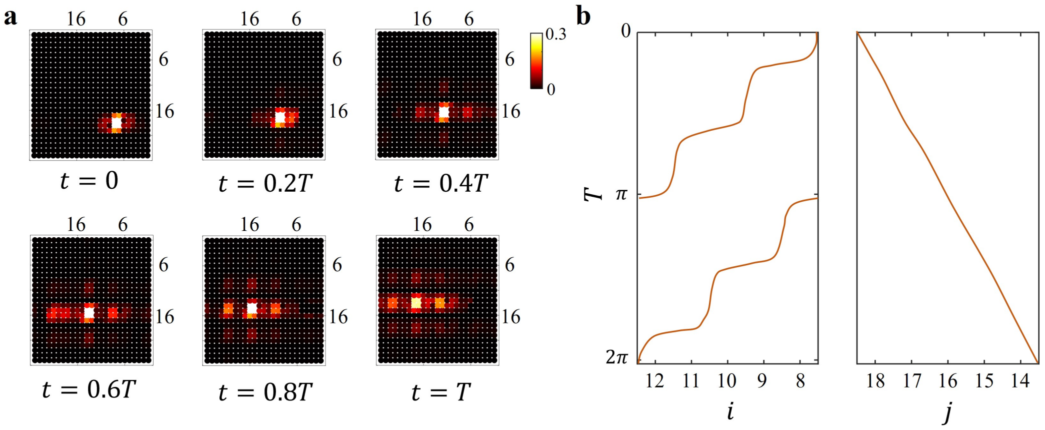


FIG. S7. Numerical results of integer-quantized Thouless pumping with distinct displacement magnitudes along orthogonal spatial directions in linear regime. (a). Spatial distributions of the wavefunction in the linear limit at six equally spaced time points during one driving period: $t = 0,\ 0.2T,\ 0.4T,\ 0.6T,\ 0.8T$, and $T$. (b). Corresponding trajectory of the wavefunction's c. m. over one complete driving cycle.

## IX. NUMERICAL RESULTS FOR THE MODIFIED 2D THOULESS PUMPING MODEL.

Since resistor-capacitor (RC) circuits can only map to lattice models with purely imaginary couplings and onsite potentials, the model in Figure 1 of the main text cannot be directly implemented using RC circuits. In this section, we design a modified 2D Thouless pumping model with purely imaginary matrix elements, which shares the same eigenenergy spectrum as the original model (Figure 1a in the main text). Moreover, by tuning the nonlinear strength, this modified model can also exhibit the same Thouless pumping behaviors governed by high-order Chern numbers.

First, the tight-binding Hamiltonian of the 2D nonlinear Thouless pumping model in momentum space, as described in the main text, can be expressed as:

$$H_{2D} = \sum_{i,j} \left[X_{i,j} a_{i,j}^{+} a_{i+e_x,j} + Y_j(t) a_j^{+} a_{j+e_y}\right] + h.c. - g\left|\phi_{i,j}(t)\right|^2, \tag{S15}$$

where $X_{i,j} = J_x + K_x cos(2\pi[\alpha_{xw}(i-1) + B_{wy}(j-1)] - \varphi_w)$ denotes the position-dependent coupling along the x-axis at sublattice $(i,j)$, with $i \in [1,5]$ and $j \in [1,5]$ indexing the sublattice sites. $Y_j(t) = J_y + K_y cos\left(2\pi[\alpha_{yu}(j-1)] - \varphi_u(t) + \alpha_0\right)$ describes the time- and position-dependent coupling along the $y$-axis. $\phi_{i,j}(t)$ represents the wavefunction amplitude localized at sublattice $(i,j)$ at time $t$, and $g$ denotes the nonlinear strength. Then, we consider a block-diagonal Hamiltonian

$$\widetilde{H}_{2D} = \begin{pmatrix} H_{2D} & 0 \\ 0 & -H_{2D} \end{pmatrix}, \tag{S16}$$

where:

$$H_{2D} = \sum_{i,j} \left[X_{i,j} a_{i,j}^{+} a_{i+e_x,j} + Y_j(t) a_j^{+} a_{j+e_y}\right] + h.c. - g\left|\phi_{i,j}(t)\right|^2. \tag{S17}$$

We perform a unitary transformation of Eq. (S17) with

$$U = \frac{1}{\sqrt{2}}\begin{pmatrix} 1 & -i \\ -i & 1 \end{pmatrix} \tag{S18}$$

Accordingly, the modified Hamiltonian can be written as

$$H_{2D}^{mod.} = U\widetilde{H}_{2D}U^{\dagger} = i\begin{pmatrix} 0 & H_{2D} \\ -H_{2D} & 0 \end{pmatrix}$$

which is a purely imaginary matrix. In this case, the time-dependent Schrödinger equation for the lattice model can be written as

$$i\frac{\partial}{\partial t}\begin{pmatrix} \phi_{i,j}(t) \\ \phi_{i',j'}(t) \end{pmatrix} = i\begin{pmatrix} 0 & H_{2D} \\ -H_{2D} & 0 \end{pmatrix}\begin{pmatrix} \phi_{i,j}(t) \\ \phi_{i',j'}(t) \end{pmatrix}. \tag{S19}$$

Here $\phi_{i',j'}(t)$ denote time-domain eigenstates localized at lattice site $(i',j')$. Expanding Eq. (S19) yields

$$i\frac{\partial}{\partial t}\phi_{i,j}(t) = iX_{i,j}\phi_{i'+1,j'}(t) + iX_{i-1,j}\phi_{i'-1,j'}(t) + iY_j(t)\phi_{i',j'+1}(t) + iY_{j-1}(t)\phi_{i',j'-1}(t)$$

$$-ig\left|\phi_{i,j}(t)\right|^2\phi_{i',j'}(t)$$

$$i\frac{\partial}{\partial t}\phi_{i',j'}(t) = -iX_{i,j}\phi_{i+1,j}(t) - iX_{i-1,j}\phi_{i-1,j}(t) - iY_j(t)\phi_{i,j+1}(t) - iY_{j-1}(t)\phi_{i,j-1}(t)$$

$$+ig\left|\phi_{i,j}(t)\right|^2\phi_{i,j}(t) \tag{S20}$$

To demonstrate that the modified model shares the same topological properties as the original model, we calculate the eigenenergy spectrum of this modified model in the linear limit as a function of $\varphi_v$, as shown in Fig. S8a. The system parameters are set as $\varphi_w = 0.8\pi$, $J_x = J_y = 0.6$, $K_x = K_y = 1$, $\alpha_{xw} = 0.2$, $\alpha_{yu} = 0.6$, $B_{wy} = 0.2$ and $\alpha_0 = 0.8\pi$. We find that this eigenenergy spectrum fully matches the linear eigenenergy spectrum of the original 2D Thouless pumping model in Fig. 1c of the main text, with the notable distinction that each point in the modified spectrum becomes doubly degenerate. Moreover, the lowest two bands exhibit second Chern numbers of $C_2^{(1)} = +2$ and $C_2^{(1)} = -3$, respectively. In fact, the eigenvalues of the modified model can be expressed as $E_{2D}^{mod.} = \begin{pmatrix} E_{2D} \\ -E_{2D} \end{pmatrix}$, where $E_{2D}$ represents the eigenvalues of the original 2D Thouless pumping model.

Next, we analyze the wave packet evolution over one period under a nonlinear strength of $g/(J_x + K_x) = 0.2$. The initial state is chosen as the modified instantaneous soliton eigenstate $\psi_{soliton}^{mod.} = \begin{pmatrix} \psi_{soliton} \\ i\psi_{soliton} \end{pmatrix}$, where $\psi_{soliton}$ is the instantaneous soliton eigenstate of the original model. Fig. S8b illustrates the spatial distribution of the wave packet at six different times: $t = 0$, $t = 0.2T$, $t = 0.4T$, $t = 0.6T$, $t = 0.8T$, and $t = T$. We observe that the modified model exhibits two independent localized wave packets at each time step, both displaying identical spatial distributions and Thouless pumping behaviors (blue and red boxes in Fig. S8(b)). The spatial distribution of the wave packet in the original 2D Thouless pumping model under the same nonlinear strength is shown in Fig. S2(c). Notably, superimposing the state distributions of the corresponding lattice sites in the blue and red boxes reproduces the wave packet distribution of the original model.

Furthermore, we compute the c. m. trajectories of the wave packets over one period for both the original and modified models, projected onto a unit cell, as depicted in Fig. S8c (blue squares for the original model and red solid lines for the modified model). The trajectories are in perfect consistency, confirming that the modified model retains the integer-quantized Thouless pumping behavior of the original model.

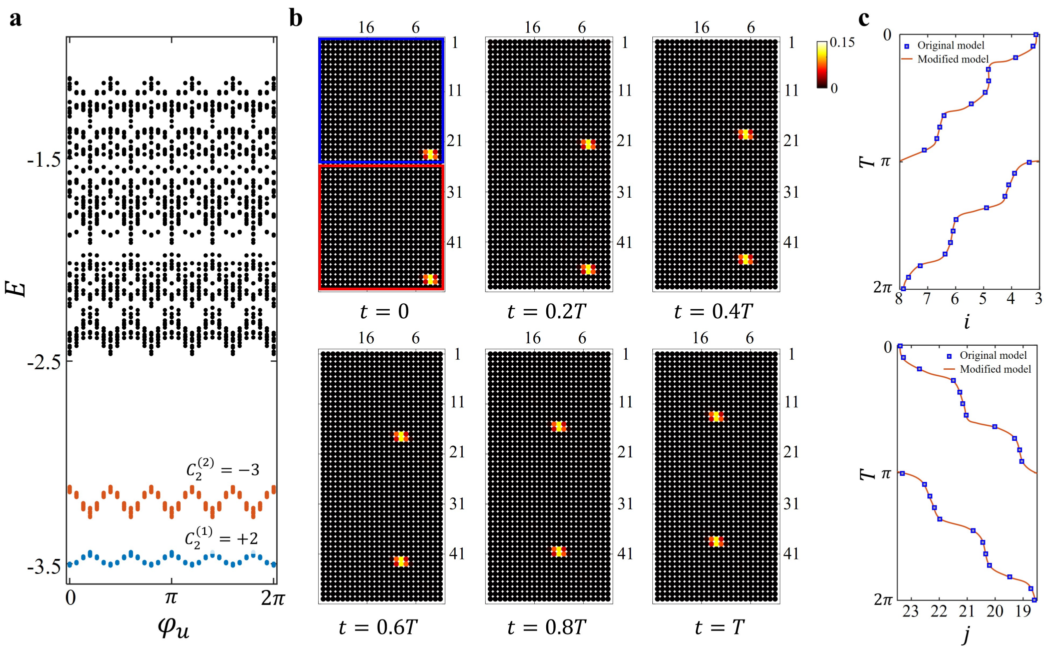


FIG. S8. Numerical results of the modified model. (a) Eigenenergy spectrum of the modified model as a function of $\varphi_v$. (b) Spatial distribution of the wavepacket at six different times $t = 0$, $t = 0.2T$, $t = 0.4T$, $t = 0.6T$, $t = 0.8T$, and $t = T$ under a nonlinear strength of $g/(J_x + K_x) = 0.2$. (c) C. m. trajectories of the wavepacket along the x- and y-axes in both the original and modified models, represented by blue squares and solid red lines, respectively.

Additionally, we compare the fractional-quantized soliton pumping behaviors of the two models under a higher nonlinear strength of $g/(J_x + K_x) = 2.8$. Figs. S9a and S9b present the spatial distributions of the wave packets at six different times for the original and modified models respectively. Similar to the integer-quantized case, the modified model maintains two identical localized wave packets throughout the evolution, both exhibiting fractional pumping. The superposition of state distributions from the blue and red boxes reproduces the original model's wave packet distribution. The c. m. trajectories of the wave packets, plotted as blue squares (original model) and red solid lines (modified model) in Fig. S9c, show excellent consistency. These results demonstrate that the modified model also exhibits the same fractional-quantized pumping behavior as the original 2D Thouless pumping model under moderate nonlinearity. It should be noted that all subsequent discussions regarding the modified model's spatial distributions refer to results obtained from the superposition of two wave packets within the blue and red boxes.

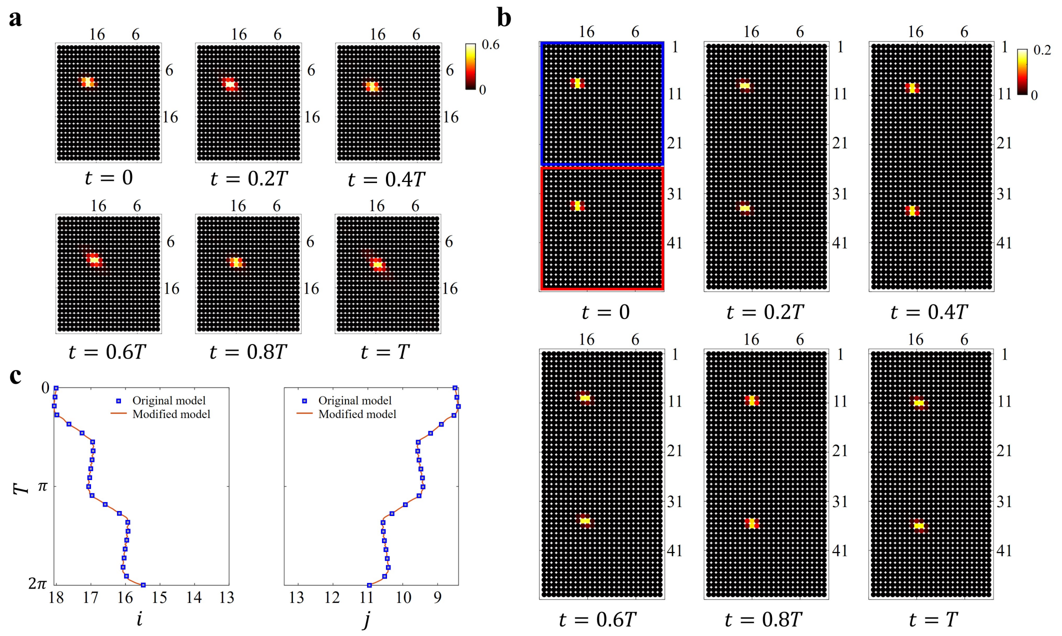


FIG. S9. Numerical results of the modified model under moderate nonlinearity. (a) and (b) Spatial distributions of wave packets in both original and modified models at six representative time instances for a nonlinear strength of $g/(J_x + K_x) = 2.8$. (c) Corresponding c. m. trajectories for the two models.

## X. THE DERIVATION OF THE TIME-VARYING ININ AND THE NONLINEAR INIC

In this section, we present the analytical derivation for the time-varying impedance converter and nonlinear impedance converter depicted in Figs. S10a and S10b.

A time-varying impedance converter comprises three conventional INICs ($\pm R$ and $\pm R_0$), two analog multipliers, and two resistors ($2R$ and $R_0$). The multiplier's transfer function follows: $V_{out}(t) = V_{in1}(t) * V_{in2}(t)/10$, where $V_{out}(t)$ denotes the output-node voltage. To achieve time-modulated INIC behavior, an external voltage signal $V(t)$ is injected into one input node of the multiplier. Applying Kirchhoff's law to the three circuit nodes labeled $V_{i,j}$, $V_0$ and $V_{i,j+1}$, we obtain three following equations as:

$$I_{i,j} = \frac{V_{i,j}-V_0}{-R} + \frac{V_{i,j}-V_{i,j+1}}{2R} \tag{S21}$$

$$I_{i,j+1} = \frac{V_{i,j+1}-V_0}{-R} + \frac{V_{i,j+1}-V_{i,j}}{2R} \tag{S22}$$

$$I_0 = \frac{V_0-V_{i,j}}{R} + \frac{V_0-V_{i,j+1}}{R} + \frac{V_0-\frac{V_{i,j}V(t)}{10}}{R_0} + \frac{V_0-\frac{V_{i,j+1}V(t)}{10}}{-R_0} \tag{S23}$$

where $I_{i,j}$, $I_0$ and $I_{i,j+1}$ correspond to currents flowing into three circuit nodes. By setting $I_{i,j}$, $I_0$ and $I_{i,j+1}$ to zero, and combing Eqs. (S21)-(S23), we can express $V_0$ as

$$V_0 = \frac{1}{2}\left(V_{i,j} + V_{i,j+1}\right) + \frac{RV(t)}{20R_0}\left(V_{i,j} - V_{i,j+1}\right). \tag{S24}$$

Then, substituting Eq. (S24) into Eqs. (S21) and (S22), we have

$$\begin{pmatrix} I_{i,j} \\ I_{i,j+1} \end{pmatrix} = \frac{V(t)}{20R_0}\begin{pmatrix} 1 & -1 \\ 1 & -1 \end{pmatrix}\begin{pmatrix} V_{i,j} \\ V_{i,j+1} \end{pmatrix} \tag{S25}$$

In this case, our designed circuit element acts as a time-varying INIC, where the current flowing into the element at the left is different from the current coming out at the right.

The proposed nonlinear impedance converter utilizes four analog multipliers, one conventional INIC ($\pm R_g$), and a resistor $R_g$. By adjusting the gain coefficients of the multipliers, the input-output relationship of each multiplier can be configured as: $V_{out}(t) = V_{in1}(t) * V_{in2}(t)$. Applying Kirchhoff's current law to the two circuit nodes labeled $V_{i,j}$ and $V_{i',j'}$ yields the two following equations:

$$I_{i,j} = \frac{V_{i,j} - V_{i,j}^2 * V_{i',j'}}{R_g}, \tag{S26}$$

$$I_{i',j'} = \frac{V_{i',j'} - V_{i,j}^3}{-R_g}, \tag{S27}$$

Eqs. (S26)-(S27) can be re-written as:

$$\begin{pmatrix} I_{i,j} \\ I_{i',j'} \end{pmatrix} = \frac{1}{R_g}\begin{pmatrix} 1 & -V_{i,j}^2 \\ V_{i,j}^2 & -1 \end{pmatrix}\begin{pmatrix} V_{i,j} \\ V_{i',j'} \end{pmatrix} \tag{S28}$$

This configuration enables the realization of a third-order nonlinear nonreciprocal coupling between nodes $(i,j)$ and $(i',j')$.

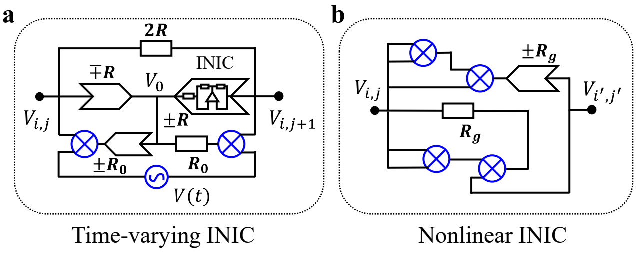


FIG. S10. The schematic diagram of (a) a time-varying INIC and (b) a nonlinear INIC.

## XI. THE DERIVATION OF THE EIGEN-EQUATION FOR THE NONLINEAR TOPOLOGICAL PUMPING CIRCUITS.

In this section, we present a detailed derivation of the eigenequation for the nonlinear topological circuit. Each circuit node $(i,j)$ is connected to five nodes: $(i',j')$, $(i',j'-1)$, $(i',j'+1)$, $(i'-1,j')$ and $(i'+1,j')$ with $i,i' \in [1,5N_x]$ and $j,j' \in [1,5N_y]$. Similarly, each circuit node $(i',j')$ is connected to five nodes: $(i,j)$, $(i,j-1)$, $(i,j+1)$, $(i-1,j)$ and $(i+1,j)$, as illustrated in Figs. S11a and S11b.

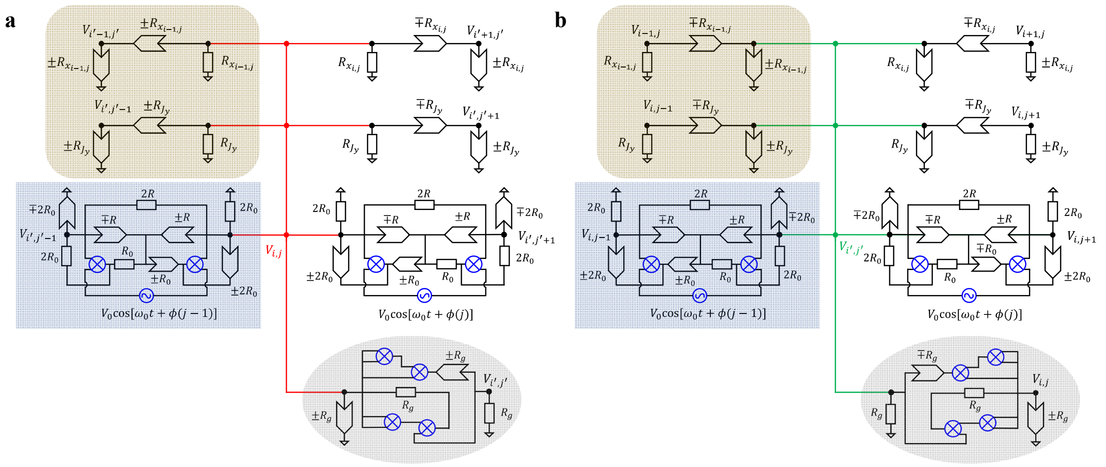


FIG. S11. Schematic diagram of the nonlinear time-modulated topolectrical circuit at nodes $(i, j)$ and $(i', j')$. Yellow, blue, and black shading correspond to constant coupling, time-varying coupling, and nonlinear coupling terms, respectively. (a) and (b) Circuit network at node $(i, j)$ and $(i', j')$.

Carrying out Kirchhoff's law on sublattice site $(i, j)$ and $(i', j')$ (there is no external source), we obtain the following equations as:

$$C\frac{d}{dt}V_{i,j} = -\frac{1}{R_{x_{i,j}}}(V_{i,j} - V_{i'+1,j'}) + \left(-\frac{1}{R_{x_{i-1,j}}}\right)(V_{i,j} - V_{i'-1,j'}) + \left(-\frac{1}{R_{J_y}} - \frac{V_0\cos[\omega_0 t + \phi(j)]}{20R_0}\right)(V_{i,j} - V_{i',j'+1})$$

$$+\left(-\frac{1}{20R_0}\right)(V_{i,j} - V_{i,j}V_0\cos[\omega_0 t + \phi(j)]) + \left(-\frac{1}{R_{J_y}} - \frac{V_0\cos[\omega_0 t + \phi(j-1)]}{20R_0}\right)(V_{i,j} - V_{i',j'-1})$$

$$+\left(-\frac{1}{20R_0}\right)(V_{i,j} - V_{i,j}V_0\cos[\omega_0 t + \phi(j)]) + \frac{1}{R_g}(V_{i,j} - V_{i',j'}^3) + \left[\frac{1}{R_{x_{i,j}}} + \frac{1}{R_{x_{i-1,j}}} + \frac{1}{R_{J_y}} + \frac{1}{20R_0} + \frac{1}{R_{J_y}} + \frac{1}{20R_0} + \frac{1}{R_g}\right]V_{i,j} \quad \text{(S29)}$$

$$C\frac{d}{dt}V_{i',j'} = \frac{1}{R_{x_{i,j}}}(V_{i',j'} - V_{i+1,j}) + \frac{1}{R_{x_{i-1,j}}}(V_{i',j'} - V_{i-1,j}) + \left(\frac{1}{R_{J_y}} + \frac{V_0\cos[\omega_0 t + \phi(j)]}{20R_0}\right)(V_{i',j'} - V_{i,j+1})$$

$$+\frac{1}{20R_0}(V_{i,j} - V_{i,j}V_0\cos[\omega_0 t + \phi(j)]) + \left(\frac{1}{R_{J_y}} + \frac{V_0\cos[\omega_0 t + \phi(j-1)]}{20R_0}\right)(V_{i',j'} - V_{i,j-1})$$

$$+\frac{1}{20R_0}(V_{i,j} - V_{i,j}V_0\cos[\omega_0 t + \phi(j)]) + \left(-\frac{1}{R_g}\right)(V_{i',j'} - V_{i,j}^3) + \left[-\frac{1}{R_{x_{i,j}}} - \frac{1}{R_{x_{i-1,j}}} - \frac{1}{R_{J_y}} - \frac{1}{20R_0} - \frac{1}{R_{J_y}} - \frac{1}{20R_0} - \frac{1}{R_g}\right]V_{i',j'} \quad \text{(S30)}$$

Eqs. (S29) and (S30) can be re-written as:

$$i\frac{d}{dt}V_{i,j} = \frac{i}{CR_{x_{i,j}}}V_{i'+1,j'} + \frac{i}{CR_{x_{i-1,j}}}V_{i'-1,j'} + \left(\frac{i}{CR_{J_y}} + i\frac{V_0\cos[\omega_0 t + \phi(j)]}{20CR_0}\right)V_{i',j'+1}$$

$$+\left(\frac{i}{CR_{J_y}} + i\frac{V_0\cos[\omega_0 t + \phi(j-1)]}{20CR_0}\right)V_{i',j'-1} - \frac{i}{CR_g}V_{i,j}^3$$

$$i\frac{d}{dt}V_{i',j'} = -\frac{i}{CR_{x_{i,j}}}V_{i+1,j} - \frac{i}{CR_{x_{i-1,j}}}V_{i-1,j} - \left(\frac{i}{CR_{J_y}}V_{i,j+1} + i\frac{V_0\cos[\omega_0 t + \phi(j)]}{20CR_0}\right)V_{i,j+1}$$

$$-\left(\frac{i}{CR_{J_y}}V_{i,j-1} + i\frac{V_0\cos[\omega_0 t + \phi(j-1)]}{20CR_0}\right)V_{i,j-1} + \frac{i}{CR_g}V_{i,j}^3, \quad \text{(S31)}$$

which exhibits the same form as Eq. (S20). The correspondence between the tight-binding model

parameters and circuit elements is given by:

$$\frac{1}{CR_{x_{i,j}}} = J_x + K_x \cos\left(2\pi\left[\alpha_{xw}(i-1) + B_{wy}(j-1)\right] - \varphi_w\right), \qquad \frac{1}{CR_{J_y}} = J_y, \qquad \frac{1}{CR_g} = g$$

$$\frac{V_0 \cos[\omega_0 t + \phi(j)]}{20CR_0} = K_y cos\left(2\pi\left[\alpha_{yu}(j-1)\right] - \varphi_u(t) + \alpha_0\right). \tag{S32}$$

Within this correspondence, the soliton pumping behavior can be visualized by tracking the spatial distribution of the node-voltage.

## XII. PROJECTION FROM A SINGLE-UNIT-CELL ONTO NEIGHBORING UNIT CELLS.

In this section, we describe the projection method used to map the spatial distribution of the voltage signal from a single-unit-cell onto the neighboring unit cells that it effectively reaches during the evolution.

In the weakly nonlinear regime ($g/(J_x + K_x) = 0.3$), we simulate the dynamics of a single-unit-cell circuit exhibiting integer-quantized pumping. The results are presented in Fig. S12a, with specific circuit parameters provided in the figure caption. Each inset shows the voltage distribution at eight equally spaced time points throughout the pumping cycle. To visualize the transport behavior more clearly, the localized voltage distributions are projected onto neighboring unit cells, as shown in the enlarged view in Fig. S12(a). Red, blue, yellow, and green boxes mark the projected positions at different spatial positions. At each time point, there is a localized voltage signal distribution, and after one full cycle, the signal shifts two unit cells along *x*- and *y*-axes, demonstrating integer-quantized pumping behavior. The c. m. trajectory of the simulation result is plotted as the black dashed line in Fig. 3c of the main text.

Fig. S12b (inset) shows the experimentally measured voltage signal distribution of the integer single-unit-cell circuit, while the projection results in the multi-unit-cell structure are shown in the enlarged panel. It can be observed that the experimentally measured voltage signals at each time point correspond well with the simulations, exhibiting integer-quantized pumping behavior. The c. m. positions are marked by red crosses in Fig. 3c of the main text.

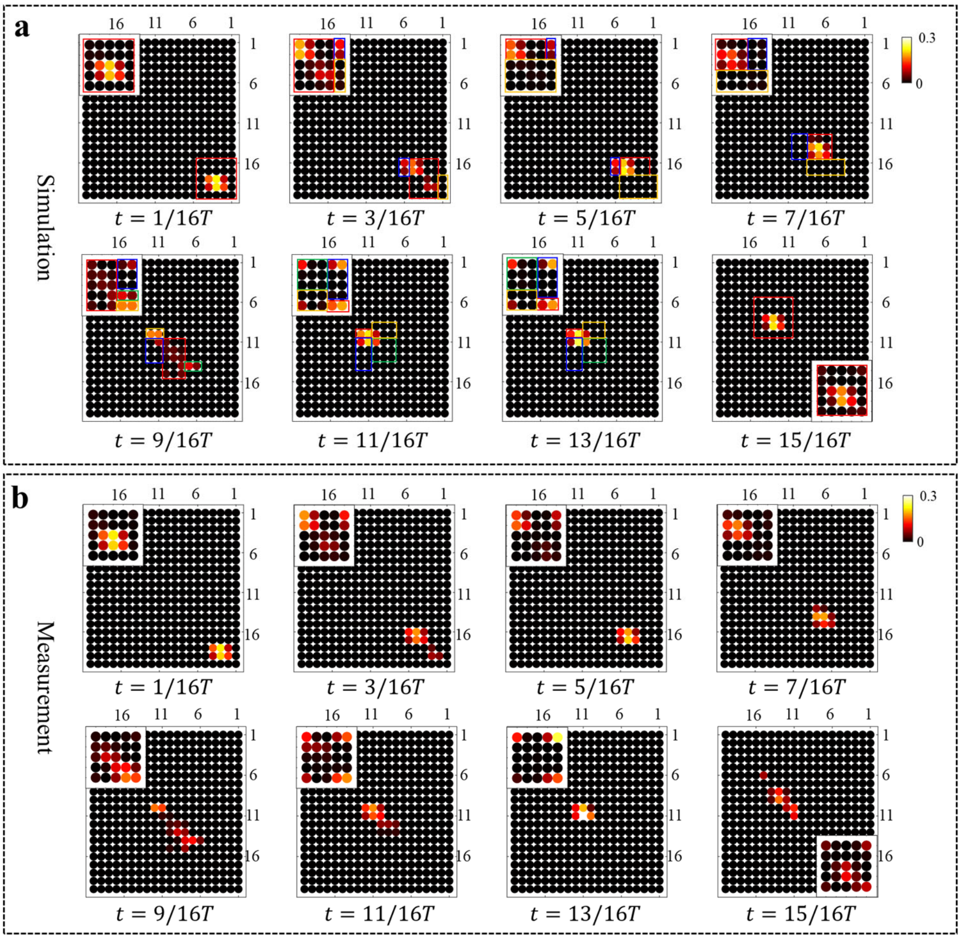


FIG. S12. Simulation and experimental results of integer single-unit-cell circuit. (a) Simulation results of the voltage signal distribution at eight different time points ($t = 1/16T$, $3/16T$, $5/16T$, $7/16T$, $9/16T$, $11/16T$, $13/16T$, and $15/16T$), shown for a single-unit-cell (inset) and multi-unit-cells (enlarged panel). Red, blue, yellow, and green boxes represent projections at different positions. The initial voltage distribution corresponds to a modified instantaneous soliton eigen-solution. The input signal at one terminal of the multiplier is a sinusoidal waveform, $V_0 \cos[\omega_0 t + \phi(j)]$, with a frequency of $\omega_0 = 11.49$ Hz. Selected component parameters include $R = 1.5\ \mathrm{k\Omega}$, $R_0 = 1.5\ \mathrm{k\Omega}$, $R_g = 6.25\ \mathrm{k\Omega}$ and $C = 100$ nf. (b) Experimental measurements of the voltage signal distribution at eight different time points, presented for a single-unit-cell (inset) and multi-unit-cells (main panel).

Furthermore, we simulate the voltage distribution of the fractional single-unit-cell circuit at five distinct time points under a nonlinear strength of $g/(J_x + K_x) = 2$, as shown in the inset of Fig. S13a. Unlike the integer circuit model, the fractional case exhibits spatial distributions that evolve in the reverse direction. The corresponding projection of the distribution onto neighboring unit cells is presented in the enlarged panel of Fig. S13(a). Evidently, the voltage signal undergoes a displacement of half a unit cell along both the $x$- and $y$-axes over one full cycle, thereby demonstrating fractional-quantized pumping

behavior. The c. m. trajectory of the simulation results is illustrated by the dashed line in Fig. 3d of the main text.

The experimental measurements of the circuit are plotted in Fig. S13b, where the inset depicts the spatial distribution of voltage signals at five different time points. The corresponding projection results in the multi-unit-cells structure are shown in the enlarged panel. It can be seen that the experimental measurements match the simulation results, also exhibiting fractional-quantized pumping behavior. The c. m. positions of the voltage signal's spatial distribution at each time point are indicated by blue crosses in Fig. 3(d) of the main text.

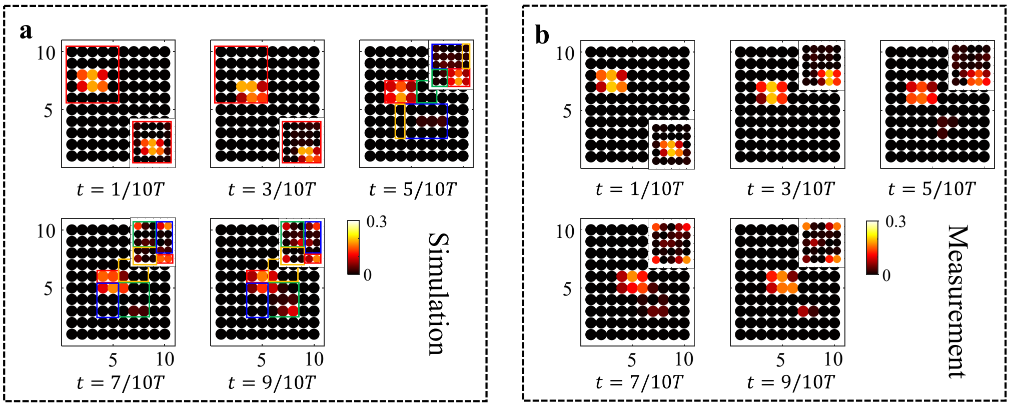


FIG. S13. Simulation and experimental results of fractional single-unit-cell circuit. (a) Simulation results of the voltage signal's spatial distribution at five different time points ($t = 1/10\,T$, $3/10\,T$, $5/10\,T$, $7/10\,T$, and $9/10\,T$), shown for a single-unit-cell (inset) and multi-unit-cells (enlarged panel). Red, blue, yellow, and green boxes represent projections at different positions. The initial voltage corresponds to a modified instantaneous soliton eigen-solution. The input frequency is $\omega_0 = 119$ Hz. Selected component parameters are $R = 1.5\ \text{k}\Omega$, $R_0 = 1.5\ \text{k}\Omega$, $R_g = 937\ \Omega$ and $C = 100$ nf. (b) Experimental measurements of the voltage signal distribution at five different time points, presented for a single-unit-cell (inset) and multi-unit-cells (enlarged panel).

## XIII. CENTER OF MASS TRAJECTORIES FROM SIMULATIONS AND EXPERIMENTS ACROSS MULTIPLE CONSECUTIVE TIME SEGMENTS.

In this section, we present a detailed comparison between circuit simulation results and experimental measurements over multiple consecutive time segments.

For the integer unit cell circuit, the driving period $T$ is partitioned into eight consecutive intervals: $t = nT/8$ to $(n+1)T/8$ ($n = 0,1,\ldots,7$). The initial voltage distributions and driving phases for each segment correspond to simulation results at the discrete time points $t = nT/8$.

The red scatter points in Figs. S14a-S14h depict the experimentally measured c. m. trajectories of the voltage signal within each interval, while the solid blue lines show the corresponding simulation results. As evident, within each segment, the experimentally measured and simulated c. m. trajectories exhibit excellent correspondence, validating the segmental measurement approach under practical constraints.

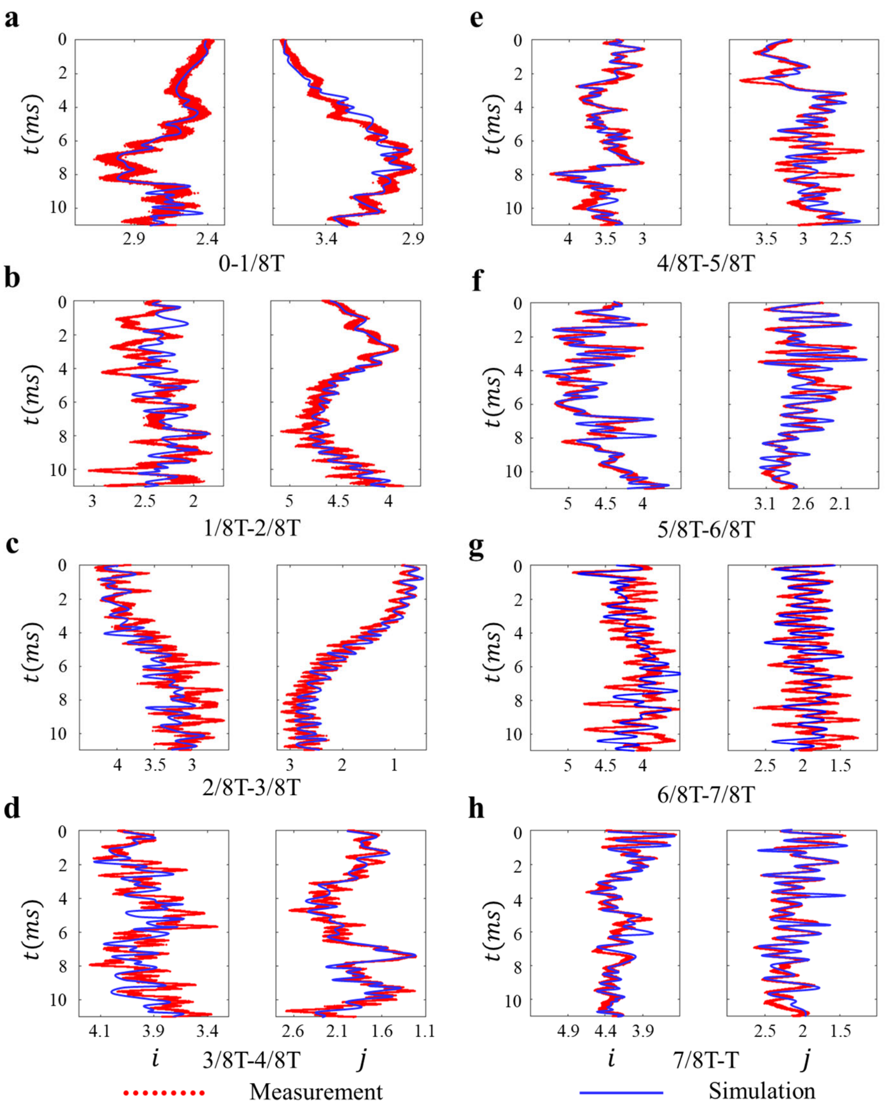


FIG. S14. Simulation and experimental results of the integer single-unit-cell circuit over multiple time intervals. (a)-(h) C. m. evolution trajectories of the voltage signal's spatial distribution across eight consecutive intervals: $t = nT/8$ to $(n+1)T/8$ $(n = 0,1,\dots,7)$. Red scatter points represent experimental measurements, and blue solid lines denote simulation results.

Furthermore, for the fractional unit-cell circuit, the driving period is divided into five consecutive

intervals: $t = nT/5$ to $(n+1)T/5$ ($n = 0,1,\dots,4$). The c. m. trajectories of the voltage signal across these intervals are plotted as red dots in Figs. S15a-S15e. Similarly, the initial voltage configuration and driving phases for each interval are set according to the simulation results at $t = nT/5$. The solid blue lines represent the corresponding simulated c. m. trajectories. The experimentally measured c. m. trajectories closely follow the simulation results across all segments, confirming the validity of the segmented measurement method for the fractional circuit configuration.

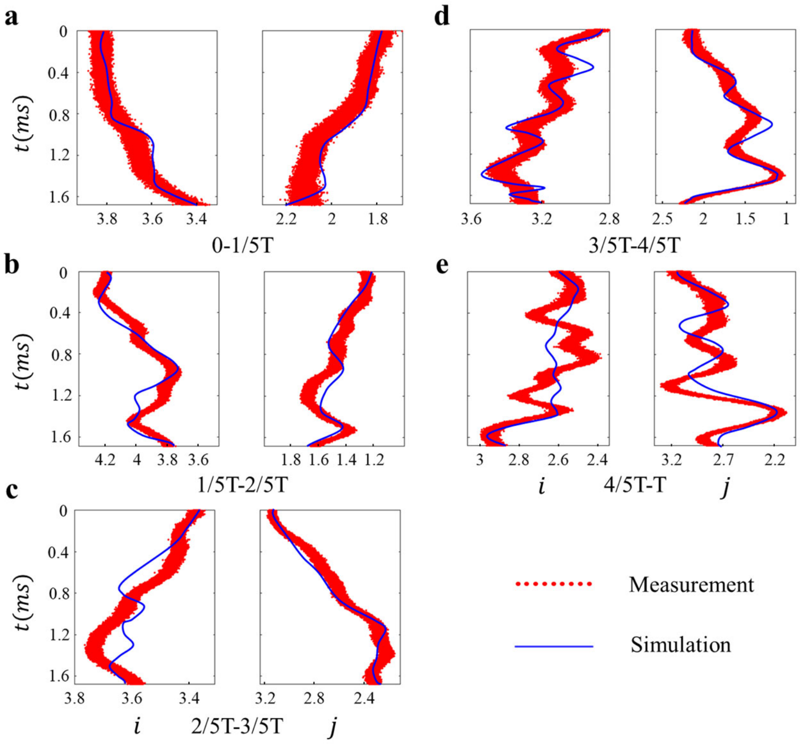


FIG. S15. Simulation and experimental results of the fractional single-unit-cell circuit across multiple time intervals. (a)-(e) C. m. trajectories of the voltage signal's spatial distribution over five consecutive intervals: $t = nT/5$ to $(n+1)T/5$ ($n = 0,1,\dots,4$). Red scatter points indicate experimental measurements, while blue solid lines represent simulation results.

## XIV. THE INFLUENCE OF RESISTIVE AND CAPACITIVE DISORDER ON THE INTEGER QUANTIZED SOLITON TRANSPORT IN CIRCUITS.

In this section, we study the influence of resistive and capacitive disorder on the integer quantized soliton transport in circuits.

We introduce disorder by adding random fluctuations to the resistors and capacitors, $R = R_0 + \delta R$ and $C = C_0 + \delta C$, where $R_0$ and $C_0$ are the disorder-free values, and $\delta R \in [-W_R R_0, W_R R_0]$, $\delta C \in$

$[-W_C C_0, W_C C_0]$. The dimensionless parameters $W_R$ and $W_C$ quantify the disorder strengths. Fig. S16a shows the circuit-simulation results for the spatial distribution of the node-voltage signals and the corresponding c. m. trajectory over one period at $W_R = 0.001$ and $W_C = 0.001$. It is clear that, at this disorder level, the node-voltage signal propagates forward as a localized spatial profile, and its centroid advances by integer lattice constants along both *x*- and *y*-axes, in agreement with the theoretical model.

To demonstrate the impact of resistance disorder on integer transport, we fix the capacitance disorder strength at $W_C = 0.001$ and simulate the circuit dynamics for two resistance-disorder values, $W_R = 0.005$ and $W_R = 0.01$. The corresponding transport behaviors are shown in Fig. S16b and Fig. S16c (red boxed regions). The results indicate that, as $W_R$ increases, the initially localized node-voltage distribution gradually spreads outward during the time evolution, and the c. m. position correspondingly deviates from the quantized trajectory. This occurs because stronger resistance disorder modifies the inter-node internal coupling and the effective nonlinear coupling, making the circuit connectivity irregular and random. The phase coherence of the node voltages is degraded, energy gradually leaks to neighboring nodes, and the localized voltage profile therefore spreads outward during the evolution.

To further assess the role of capacitance disorder, we fix the resistance disorder strength at $W_R = 0.001$ and simulate the spatial distributions of the node-voltage signals together with their corresponding c. m. trajectories for $W_C = 0.01$, $W_C = 0.05$, and $W_C = 0.1$. The results are shown in Figs. S16d-S16f (blue boxed regions). It is evident that, compared with resistance disorder—which perturbs the internal inter-node coupling and the effective nonlinear coupling—ground-capacitance disorder has a much weaker impact on the transport of a localized voltage signal. For disorder levels below $0.01$, the system still exhibits well-preserved integer transport.

These results indicate that the topological pumping behavior in our circuit is highly sensitive to resistance disorder and should be kept within 0.001, whereas the influence of capacitance disorder on voltage-signal transport is comparatively small (acceptable when within 0.01). Moreover, for circuit configurations exhibiting fractional transport, we observe similar trends under the same resistance and capacitance disorder levels. Therefore, for practical circuit experiments aiming at stable integer and fractional quantized transport, it is advisable to keep both the resistance and capacitance disorder within 0.001.

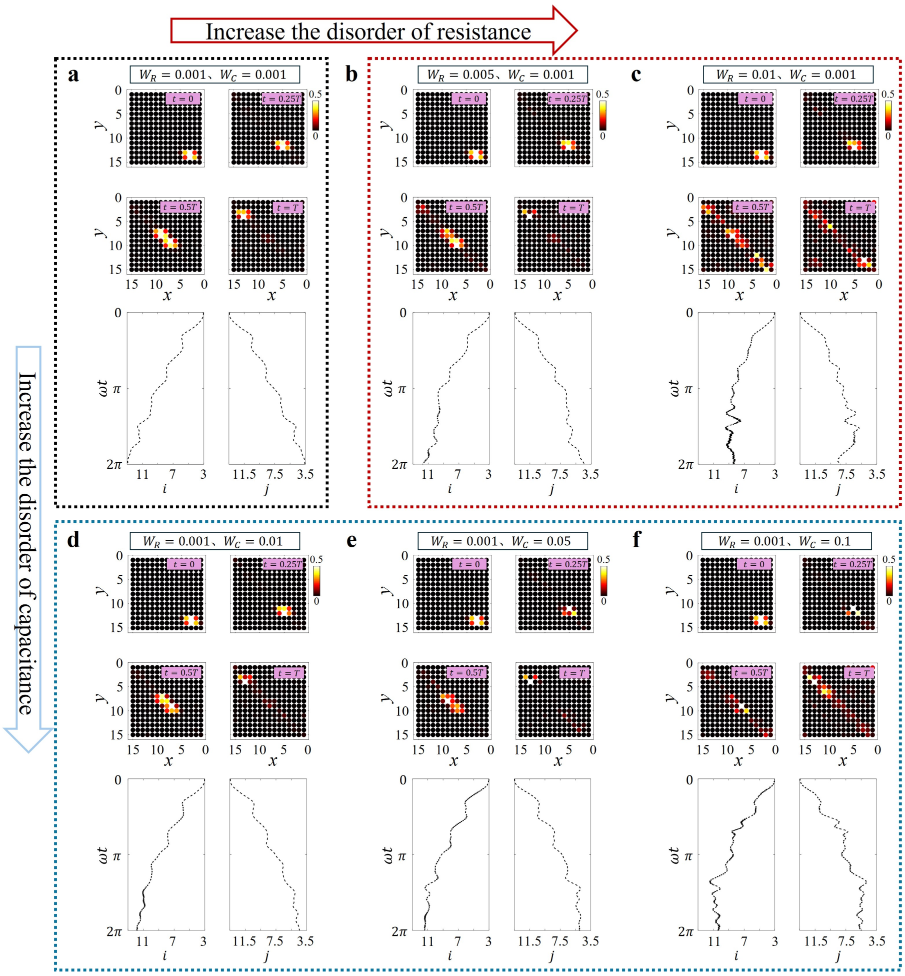


FIG. S16**.** Circuit-simulation results showing the spatial distributions of node-voltage signals and the corresponding c. m. trajectories under different resistance and capacitance disorder strengths. (a) $W_R = 0.001$ and $W_C = 0.001$: top, instantaneous spatial distribution of node voltages; bottom, c. m. trajectory over one pumping period. (b) and (c) With capacitance disorder fixed at $W_C = 0.001$, results for resistance disorder $W_R = 0.005$ and $0.01$. (d)-(f). With resistance disorder fixed at $W_R = 0.001$, results for capacitance disorder $W_C = 0.01, 0.05,$ and $0.1$.

## XV. NUMERICAL RESULTS OF 3D INTEGER-INTEGER-INTEGER AND FRACTIONAL-FRACTIONAL-FRACTIONAL THOULESS PUMPING OF SOLITONS GOVERNED BY FIRST, SECOND, AND THIRD CHERN NUMBERS.

Beyond the 2D nonlinear Thouless pumping model, we extend our investigation to a three-dimensional (3D) nonlinear Thouless pumping system with a unit cell composed of $5 \times 5 \times 5 = 125$ sublattices, as illustrated in Fig. S17a. Similar to the 2D case, this model consists of two key components: (i) a linear coupling term and (ii) an onsite Kerr nonlinearity (denoted by gray dots). The linear part is constructed from three independent AAH models, each embedded in orthogonal 2D subspaces — $(x, w)$,

$(y, u)$, and $(z, v)$ — where $x$, $y$, $z$ denote spatial dimensions, while $w$, $u$, $v$ represent the synthetic dimensions. Within each of these subspaces ($xw$, $yu$, and $zv$), every plaquette is threaded by an intrinsic magnetic flux, denoted as $\alpha_{xw}$, $\alpha_{yu}$, and $\alpha_{zv}$, respectively. The nearest-neighbor coupling includes both a constant term $J_{x,y,z}$ and a modulated term $K_{x,y,z}$.

The connection between third Chern numbers and soliton pumping can be established by introducing an additional magnetic field perturbation $B_{uz}$ in the $uz$-plane, extending the 2D framework. The electric field along the $u$-axis is replaced by an $E_v$-field along the $v$-axis, ensuring the adiabatic and periodic evolution of the phase $\varphi_v(t)$. Under these conditions, the coupling along the $x$-axis at sublattice $(i, j, k)$ is given by $X_{i,j,k} = J_x + K_x cos(2\pi[\alpha_{xw}(i-1) + B_{wy}(j-1)] - \varphi_w)$, which depends on the horizontal ($i$), vertical ($j$), and longitudinal ($k$) axes. The coupling along the $y$-axis at the sublattice $(i, j, k)$ takes the form: $Y_{i,j,k} = J_y + K_y cos\big(2\pi[\alpha_{yu}(j-1) + B_{uz}(k-1)] - \varphi_u\big)$, where it varies with both the vertical ($j$) and longitudinal ($k$) axes. The time-dependent coupling along $z$-axis, $Z_j(t) = J_z + K_z cos(2\pi[\alpha_{zv}(k-1)] - \varphi_v(t) + \alpha_0)$, depends exclusively on the longitudinal axis ($k$).

The nonlinear term originates from the onsite Kerr nonlinearity $g\left|\phi_{i,j,k}(t)\right|^2$, where $g$ represents the nonlinear strength and $\phi_{i,j,k}(t)$ denotes the wavefunction amplitude at sublattice $(i, j, k)$ and time $t$. By combining the linear and nonlinear contributions, we obtain the 3D nonlinear time-domain dynamical equation for the system:

$$i\frac{\partial}{\partial t}\phi_{i,j,k}(t) = X_{i,j,k}\phi_{i+1,j,k}(t) + X_{i-1,j,k}\phi_{i-1,j,k}(t) + Y_{i,j,k}\phi_{i,j+1,k}(t) + Y_{i,j-1,k}\phi_{i,j-1,k}(t)$$

$$+Z_k(t)\phi_{i,j,k+1}(t) + Z_{k-1}(t)\phi_{i,j,k-1}(t) - g\left|\phi_{i,j,k}(t)\right|^2\phi_{i,j,k}(t). \qquad \text{(S33)}$$

Fig. S17b presents the system's linear band structure as a function of $k_y$ and $k_z$ over the range [$-\pi$, $\pi$], where ground states, first excited states, and higher-energy states are marked by blue, red, and black dots respectively. Detailed parameters are provided in the figure caption. We can see that the two lowest bands are well isolated from higher bands by a substantial gap. In Fig. S17c, eigenenergy spectra are plotted as functions of $\varphi_v$, obtained through momentum-space scans for each $\varphi_v$. It is noted that the two lowest bands maintain their non-degenerate character and remain energetically isolated from higher bands throughout the evolution, confirming the stability of the system's topological properties during temporal dynamics. Furthermore, we calculate the nontrivial third Chern numbers $\{C_3^{(1)} = -2, C_3^{(2)} = +3\}$ for the lowest two bands in the 6D parameter space $(k_x, k_y, k_z, \varphi_w, \varphi_u, \varphi_v)$, which quantitatively characterizes their topological nature.

In the previous discussion in the main text, we explored how varying the strength of nonlinearity in the 2D off-diagonal model can achieve diverse quantized pumping transport behaviors. In this section, we extend our investigation to the 3D off-diagonal model, examining how nonlinearity and higher-order Chern number interactions influence the transport dynamics of solitons. First, under weak nonlinearity $g/(J_x+K_x)=0.5$, we calculate the time evolution of a wave packet over one driving period $T$. Fig. S17d1 shows the spatial distribution of the wave packet at three distinct times: $t=0$, $t=0.5T$, and $t=T$, represented by red squares, blue circles, and green triangles, respectively. The initial wave packet is an instantaneous soliton eigen-solution. Compared to the 2D model, the wave packet exhibits integer-integer-integer soliton transport with identical displacement magnitudes along all three spatial axes, governed by distinct-order Chern numbers. After one pumping cycle, the wave packet shifts by $C_3^{(1)}=-2$, $C_2^{(1)}=+2$ and $C_1^{(1)}=-2$ unit cells along the $x$-, $y$-, and $z$-axes, respectively, while preserving its initial spatial profile.

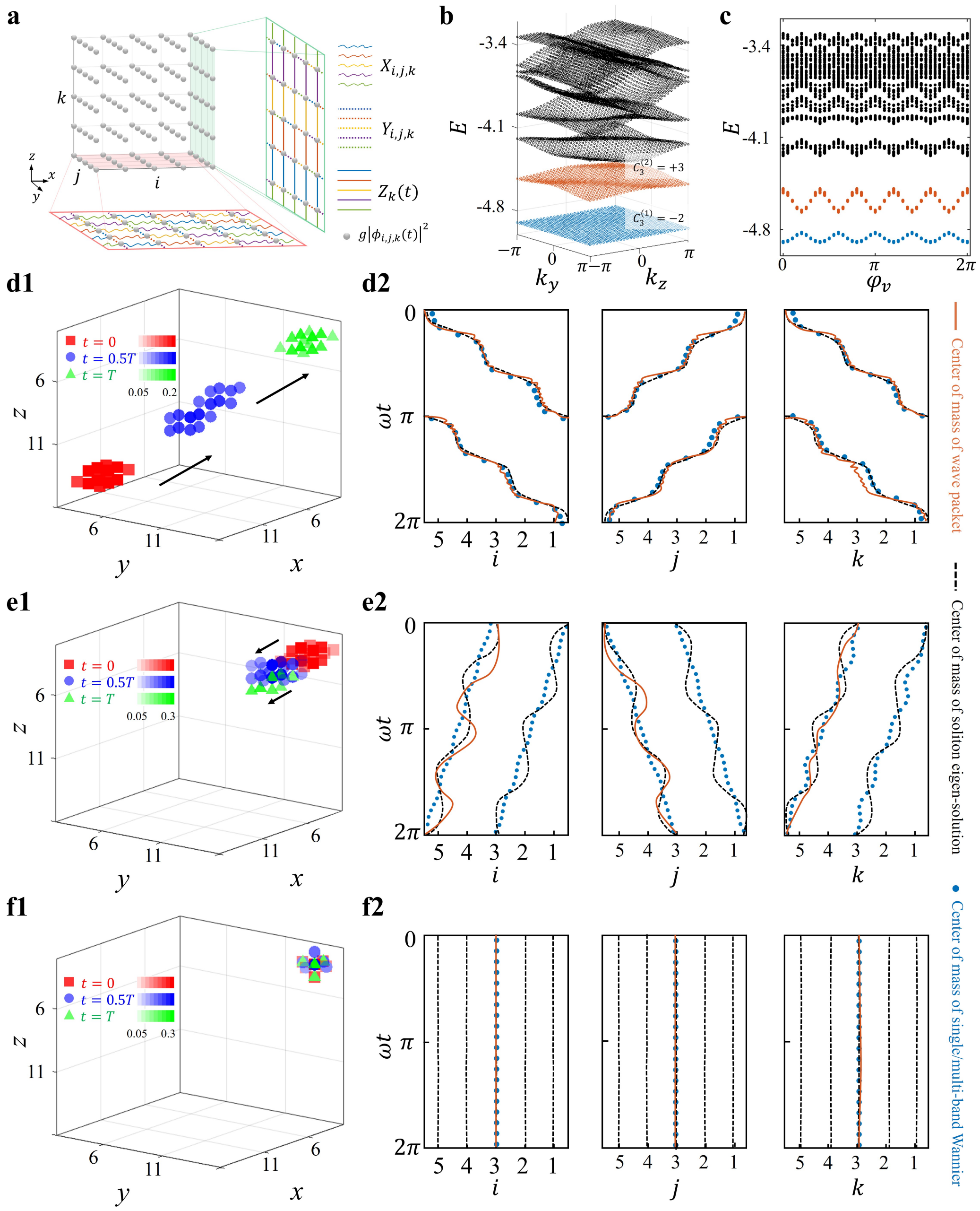


FIG. S17. Theoretical results of 3D nonlinear Thouless pumping dictated by first, second, and third Chern numbers. (a) Schematic of a 3D Thouless pumping model with 125 sublattices per unit cell, incorporating on-site Kerr nonlinearity. The red and green solid boxes depict the intracell couplings ($X_{i,j,k}$, $Y_{i,j,k}$, $Z_k(t)$) on the $x$-$y$ and $y$-$z$ planes, respectively, along with the nonlinear term ($g|\phi_{i,j,k}|^2$). Curves of different colors, along with dashed and solid lines, represent intracell couplings along different directions, while gray dots denote the on-site potential term, as shown in the right inset. (b) Low-energy bands of the linear Hamiltonian in the 6D parameter space ($k_x, k_y, k_z, \varphi_w, \varphi_u, \varphi_v$), plotted as a function of $k_y$ and $k_z$ over $[-\pi, \pi]$. The ground state (third Chern number $C_3^{(1)} = -2$) and the first excited state ($C_3^{(2)} = +3$) are marked by blue and red dots, respectively; higher-energy

states are shown in black. System parameters are set as $k_x = 0$, $\varphi_w = 0.8\pi$, $\varphi_u = 0.6\pi$, $\varphi_v = 0$, $J_x = 0.55$, $J_y = 0.4$, $J_z = 0.55$, $K_x = K_y = K_z = 1$, $\alpha_{xw} = 0.2$, $\alpha_{yu} = 0.2$, $\alpha_{zv} = 0.6$, $B_{wy} = 0.2$, $B_{uz} = 0.2$, and $\alpha_0 = 1.6\pi$. (c) Low-energy bands over a full cycle of $\varphi_v$, with a full momentum-space scan performed for each $\varphi_v$. (d1) -(f1) Wave-packet spatial distributions at nonlinearity strengths $g/(J_x + K_x) = 0.5$, 3, and 6. Red squares, blue circles, and green triangles correspond to times $t = 0$, $t = 0.5T$, and $t = T$, respectively. The modulation frequency is $\omega = \partial\varphi_v/\partial t = 2\pi/500$. (d2) -(f2) C. m. trajectories along the $x$-, $y$- and $z$-axes. The wave packet, instantaneous soliton, and single-/multi-band Wannier functions are represented by orange solid lines, black dashed lines, and blue dots, respectively.

To verify this behavior, we compute the c. m. trajectory of the wave packet over one pumping cycle, projected onto a unit cell (Fig. S17d2). The c. m. trajectories along $x$-, $y$-, and $z$-axes (orange solid lines) closely match those of the instantaneous soliton eigenstate (black dashed lines) and the maximally localized Wannier functions (blue dots). The Wannier functions, constructed from uniform superpositions of Bloch states in the lowest band, correspond to distinct parameter spaces: $(k_z, \varphi_v)$ for $z$, $(k_y, k_z, \varphi_u, \varphi_v)$ for $y$, and $(k_x, k_y, k_z, \varphi_w, \varphi_u, \varphi_v)$ for $x$. Notably, their c. m. trajectories are quantized by the first-, second-, and third-order Chern numbers, respectively. These results demonstrate that weak nonlinearity in 3D Thouless pumping yields integer-integer-integer soliton transport dictated by distinct-order Chern numbers.

At stronger nonlinearity ($g/(J_x + K_x) = 3$), the soliton's pumping behavior changes significantly. Fig. S17e1 shows the spatial distribution at $t = 0$, $t = T$, and $t = 2T$, marked by red squares, blue circles, and green triangles, respectively. In contrast to the 3D integer Thouless pumping behavior observed under weak nonlinearity, the soliton in the moderate nonlinear regime exhibits reversed transport along all three directions by half a unit cell. Moreover, after one driving period, the soliton no longer retains the same spatial profile as the initial state. These results confirm the emergence of fractional-fractional-fractional soliton transport behavior.

This phenomenon can be elucidated through an analysis of maximally localized single- and multi-band Wannier functions. As shown in Fig. S17e2, the orange solid line, black dashed line, and blue dots respectively depict the c. m. trajectories of the wave packet, instantaneous soliton eigenstate, and single/multi-band Wannier functions. Notably, the c. m. trajectories of the instantaneous soliton exhibit remarkable consistency with those of the Wannier functions, with both sets of positions following two

distinct trajectories. The wave packet's c. m. motion follows one branch of these paths. We note that the alternative trajectory can be realized by choosing different initial eigenstates as guesses. Here, nonlinearity-induced interband coupling hybridizes the two lowest bands (originally weakly separated), necessitating a multi-band Wannier description for transport along these three axes. The c. m. positions of the multi-band Wannier functions along the $x$-, $y$-, and $z$-axes are determined by the averaged third Chern numbers $C_3^{arv} = (C_3^{(1)} + C_3^{(2)})/2 = +0.5$, averaged second Chern number $C_2^{arv} = (C_2^{(1)} + C_2^{(2)})/2 = -0.5$, and averaged first Chern number $C_1^{arv} = (C_1^{(1)} + C_1^{(2)})/2 = +0.5$. Thus, moderate nonlinearity enables exhibiting fractional-fractional-fractional quantized soliton transport, governed by distinct-order Chern numbers.

At the nonlinear strength of $g/(J_x + K_x) = 6$, the system stabilizes into a trapped soliton state (Fig. S17f1). This localized state persists even when $g/(J_x + K_x)$ exceeds 6. The spatial distributions of the wave packet at $t = 0$, $t = T$, and $t = 2T$ confirm that the wave packet remains tightly localized at its initial position throughout its evolution. To unravel this behavior, we analyze the maximally localized multi-band Wannier functions constructed from all energy bands. Their c. m. trajectories along the $x$-, $y$-, and $z$-axes are plotted as blue dots in Fig. S17f2. Notably, the c. m. displacements of these Wannier functions are governed by the summed Chern numbers: $\sum_{i=1}^{5} C_1^{(i)} = 0$, $\sum_{i=1}^{25} C_2^{(i)} = 0$, and $\sum_{i=1}^{125} C_3^{(i)} = 0$. In this regime, the Wannier functions exhibit extreme spatial localization, effectively approximating delta functions pinned to individual sublattice sites. Strikingly, the c. m. trajectories of both the wave packet (orange solid line) and the instantaneous soliton eigensolution (black dashed line) coincide perfectly with those of the Wannier functions. This remarkable consistency demonstrates that, under strong nonlinearity, the soliton's arrested transport in all three spatial directions arises from interband coupling across all Chern numbers of different orders.

## XVI. NUMERICAL RESULTS OF ANISOTROPIC INTEGER-FRACTIONAL-FRACTIONAL THOULESS PUMPING OF SOLITONS GOVERVED BY FIRST TO THIRD CHERN NUMBERS.

In this section, we present numerical demonstrations of anisotropic integer-fractional-fractional soliton pumping in a 3D Thouless pumping model, realized through precise tuning of the intrinsic magnetic fluxes. The system is configured with flux parameters $\alpha_{xw} = 1/N_x$, $\alpha_{yu} = 3/N_y$, and $\alpha_{zv} = 1/N_y$. Other system parameters are provided in the figure captions.

The linear band structure over $(k_y,\ k_z) \in [-\pi, \pi]$ is shown in Fig. S18a. The two lowest bands—marked by blue and red dots representing the ground and first excited states, respectively—carry nontrivial third Chern numbers, $C_3^{(1)} = -2$ and $C_3^{(2)} = +3$. These bands remain well separated from the higher-energy states by a substantial bandgap as $k_y$ and $k_z$ vary from $-\pi$ to $\pi$. Furthermore, we calculate the eigenenergy spectrum as a function of $\varphi_v$, as shown in Fig. S18b. The results confirm that the bandgap persists without closing, and no degeneracies occur between the lowest two bands during the evolution of $\varphi_v$, indicating the persistence of a stable topological phase.

Fig. S18c1 shows the spatial distribution of the wave packet at $t = 0,\ 0.5T$, and $T$ for weak nonlinearity ($g/(J_x + K_x) = 0.4$), initialized as an instantaneous soliton eigen-solution. The wave packet shifts by two unit cells along the $x$ and $y$ directions, and by one unit cell along $z$ per driving cycle, demonstrating integer-quantized Thouless pumping with distinct displacement magnitudes along orthogonal spatial dimensions. The c. m. trajectories, shown in Fig. S18c2 (orange solid lines), closely follow both the trajectory of the instantaneous eigenstate (black dashed lines) and the lowest-band Wannier functions (blue dots). These displacements are topologically governed by the lowest band's Chern numbers: the third Chern number $C_3^{(1)} = -2$ (x-direction), second Chern number $C_2^{(1)} = +2$ (y-direction), and first Chern number $C_1^{(1)} = -1$ (z-direction).

At moderate nonlinearity ($g/(J_x + K_x) = 4.5$, Fig. S18d1), integer-fractional-fractional Thouless pumping emerges along three orthogonal directions. The wave packet shifts by half a unit cell along the x- and y-axes, and by a full unit cell along the z-axis per driving cycle. To elucidate this behavior, we plot the c. m. trajectories of the wave packet, the instantaneous soliton eigenstate, and the single-/multi-band Wannier function, as displayed in Fig. S18d2. Notably, the c. m. trajectory along the *z*-axis is calculated for the lowest band in 2D parameter subspace $(k_z, \varphi_v)$, while those along *x*- and *y*-axes are computed for the two lowest bands in the 6D parameter space $(k_x, k_y, k_z, \varphi_w, \varphi_u, \varphi_v)$ and the 4D parameter space $(k_y, k_z, \varphi_u, \varphi_v)$. These trajectories exhibit excellent consistency, indicating that the integer-fractional-fractional soliton transport originates from interband coupling. The displacements are governed by the averaged third, second, and first Chern numbers: $C_3^{ave} = (C_3^{(1)} + C_3^{(2)})/2 = +1/2$ ($x$-direction), $C_2^{ave} = (C_2^{(1)} + C_2^{(2)})/2 = -1/2$ ($y$-direction), and $C_1^{ave} = (C_1^{(1)} + C_1^{(2)})/2 = -1$ ($z$-direction).

Under strong nonlinearity ($g/(J_x + K_x) = 10$, Fig. S18e1), the system transitions into a trapped soliton regime. As shown in Fig. S18e2, the c. m. trajectories of both the wave packet and the

instantaneous soliton eigenstate closely follow those of the maximally localized multi-band Wannier functions calculated across all bands. In this case, the system exhibits the same Thouless pumping behavior as the first intrinsic flux configuration under strong nonlinearity. The displacements along the $x$-, $y$-, and $z$-axes are determined by the averaged third-, second-, and first-order Chern numbers, respectively: $C_3^{ave} = \frac{\sum_{i=1}^{125} C_3^{(i)}}{5} = 0$, $C_2^{ave} = \frac{\sum_{i=1}^{25} C_2^{(i)}}{25} = 0$, and $C_1^{ave} = \frac{\sum_{i=1}^{5} C_1^{(i)}}{125} = 0$. These vanishing averaged Chern numbers confirm the absence of transport in the strong nonlinearity regime, consistent with soliton localization.

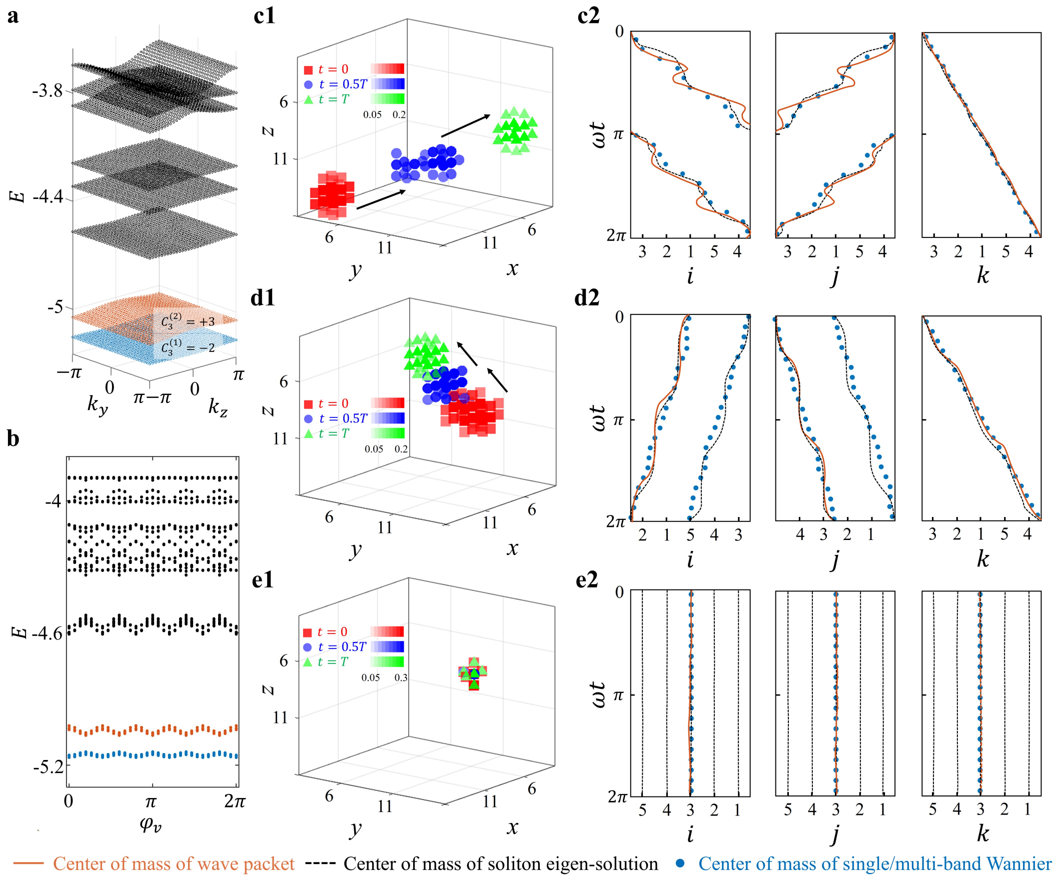


FIG. S18. Theoretical results of 3D nonlinear Thouless pumping dictated by high-order Chern numbers. (a) Band structure of the 3D Thouless pumping model with intrinsic magnetic fluxes $\alpha_{xw} = 0.2$, $\alpha_{yu} = 0.6$, and $\alpha_{zv} = 0.2$. Other system parameters are $k_x = 0$, $\varphi_w = 0.2\pi$, $\varphi_u = \pi$, $\varphi_v = 0$, $J_x = 0.35$, $J_y = 0.45$, $J_z = 0.9$, $K_x = K_y = K_z = 1$, $B_{wy} = 0.2$, $B_{uz} = 0.2$, and $\alpha_0 = 1.6\pi$. (b) Numerical results of the eigenenergy spectra over a full cycle of $\varphi_u$. (c1), (d1), and (e1) The spatial distributions of the wave packet at three characteristic time points ($t = 0$, $0.5T$, and $T$) for varying nonlinearity strengths ($g/(J_x + K_x) = 0.4$, $4.5$, and $10$). The corresponding c. m. trajectories of wave packets are plotted as orange solid lines in panels (c2), (d2), and (e2) the trajectories of instantaneous soliton eigen-solution and maximally localized single-/multi-band Wannier functions are represented

by black dashed lines and blue dots respectively.

## XVII. CIRCUIT SIMULATION RESULTS OF THE 3D ANISOTROPIC INTEGER-FRACTIONAL-FRACTIONAL THOULESS PUMPING MODEL.

In this section, we present the circuit simulation results of the 3D nonlinear Thouless pumping model. Similar to the 2D nonlinear Thouless pumping model, to enable simulation using resistor-capacitor (*RC*) circuits, we designed a modified version of the 3D nonlinear Thouless pumping model with purely imaginary matrix elements. This model shares the same energy spectrum and Thouless pumping behaviors governed by higher-order Chern numbers as the original model. For the modified model, we constructed a 3D circuit model primarily composed of three modules: conventional INICs, time-varying INICs, and nonlinear INICs.

Figs. S19a and S19b illustrate the network structures at circuit nodes $(i, j, k)$ and $(i', j', k')$, respectively. Each node is connected to six conventional INICs, two time-varying INICs, and one third-order nonlinear INIC, where $i, i' \in [1, 5N_x]$, $j, j' \in [1, 5N_y]$ and $k, k' \in [1, 5N_z]$. $N_x$, $N_y$ and $N_z$ represent the number of unit cells along the $x$-, $y$- and $z$-axes.

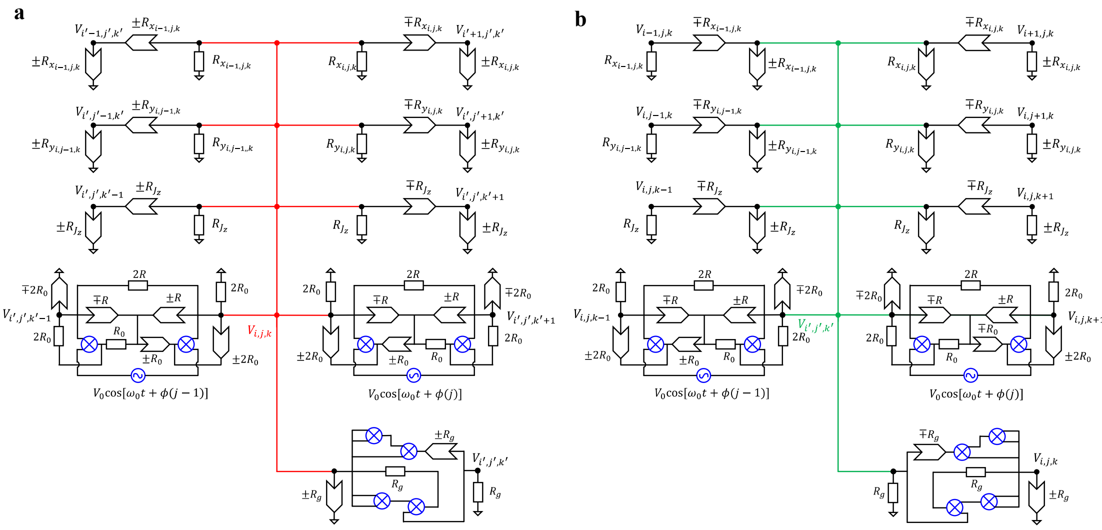

FIG. S19. Schematic diagram of the 3D nonlinear topological pumping circuit at circuit nodes $(i, j, k)$ and $(i', j', k')$. (a) Circuit network at node $(i, j, k)$. (b) Circuit network at node $(i', j', k')$.

We first simulated the integer-quantized pumping model with a nonlinear strength of $g/(J_x + K_x) = 0.4$. Fig. S20a shows the voltage distributions at five different time points ($t = 0, 0.25T, 0.5T, 0.75T, \text{and } T$) in the 3D single-unit-cell circuit model. The voltage signal exhibits clear quantized shifts of $-2a$, $+2a$, and $-a$ along the $x$-, $y$-, and $z$-axes, respectively, during temporal

evolution, and returns to its initial spatial configuration after one full cycle. The projected c. m. trajectories along the three orthogonal axes are shown as black dashed lines in Fig. S20b, exhibiting precise consistency with the theoretically calculated trajectories (solid red lines).

Next, we simulated the fractionally quantized pumping model with a nonlinear strength of $g/(J_x + K_x) = 4$. The voltage distributions at five representative time points are illustrated in Fig. S20c. During each pumping cycle, the voltage signal exhibits fractional displacements of $+1/2a$, $-1/2a$, and $-a$ along the $x$-, $y$-, and $z$-axes, respectively. To better visualize the signal dynamics, we further plotted the projected c. m. trajectories along the three orthogonal directions as black dashed lines in Fig. S20d. The theoretically calculated c. m. trajectories for the corresponding fractional pumping model are shown as solid red lines, exhibiting excellent agreement.

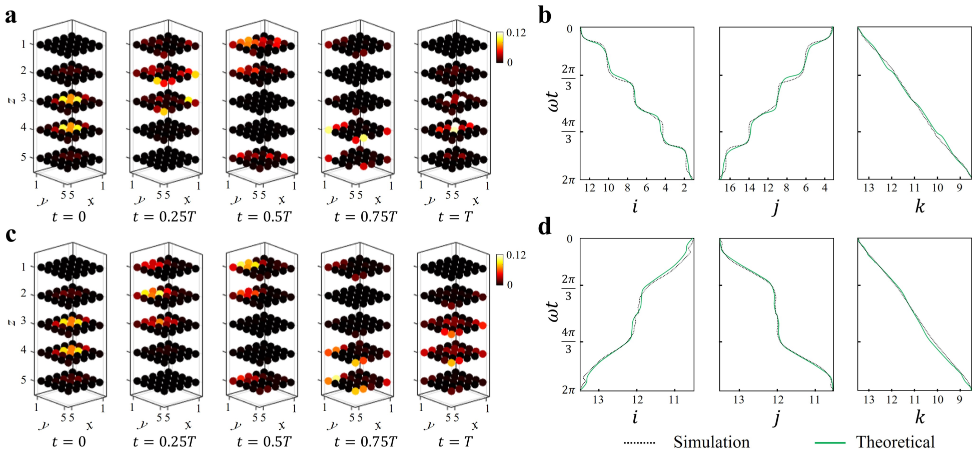


FIG. S20. Circuit simulation results of the 3D nonlinear Thouless pumping model. (a) Voltage spatial distributions of the 3D integer unit-cell circuit at five time points ($t = 0, 0.25T, 0.5T, 0.75T, T$). Circuit parameters: $R = 1.5$ kΩ, $R_g$=5.56 kΩ, $\omega = 13.33$ Hz, $V_0 = 10$ V. (b) Projected c. m. trajectories along the x-, y- and z-axes, with black dashed lines and solid red lines representing circuit simulation results and theoretical calculations, respectively. (c) Voltage spatial distributions of the 3D fractional unit-cell circuit at five time points. Circuit parameters: $R = 1.5$ kΩ, $R_g$=0.556 kΩ, $\omega = 41.67$ Hz, $V_0 = 10$ V. (d) Corresponding projected c. m. trajectories.

## XVIII. THE QUANTIZED TRANSPORT OF WAVE PACKETS WITH A SATURABLE NONLINEARITY

In this section, we analyze soliton transport with a saturable nonlinearity. Specifically, we replace the Kerr term $-g_s|\phi|^2\phi$ by a saturable nonlinearity

$$f_{sat}(|\phi|^2\phi) = -g_s \frac{|\phi|^2}{1 + s|\phi|^2}\phi, \tag{S34}$$

where $s$ is the saturation parameter. The evolution equation of the modified model becomes

$$i\frac{\partial}{\partial t}\phi_{i,j}(t) = X_{i,j}\phi_{i+1,j}(t) + X_{i-1,j}\phi_{i-1,j}(t) + Y_j(t)\phi_{i,j+1}(t) + Y_{j-1}(t)\phi_{i,j-1}(t)$$
$$-g_s \frac{|\phi_{i,j}(t)|^2}{1 + s|\phi_{i,j}(t)|^2}\phi_{i,j}(t) \tag{S35}$$

For comparison, Figs. S21a and S21b display the spatial profiles of the wave packet and the corresponding c. m. trajectories for the linear and Kerr-nonlinearity cases at $t = 0$, $t = 0.5T$, and $t = T$. Furthermore, for the model with saturable nonlinearity, we fix the nonlinear coefficient at $g_s = 0.2(J_x + K_x)$ and compute the spatial distributions and the corresponding c. m. trajectories for $s = 10, 20, 50,$ and $80$, as shown in Figs. S21c-S21f. We can see that, for the smaller values $s = 10$ and $20$, integer-quantized soliton transport still occurs. For the larger values $s = 50$ and $80$, the packet no longer remains localized as a soliton during propagation but instead gradually spreads outward, exhibiting transport behavior similar to the linear case.

This is because, with $g_s$ fixed, when $s$ is small the nonlinearity does not enter saturation within the intensity range of our model, and $f_{sat}(|\phi|^2\phi) \approx -g_s|\phi|^2\phi$. The system is then in a Kerr-type strong-nonlinearity regime, capable of forming solitons and exhibiting integer soliton transport. However, increasing $s$ causes the nonlinearity to saturate at lower intensities, which significantly weakens the nonlinear effect. In the limit $s \to \infty$, the nonlinearity becomes nearly constant, making it difficult to sustain solitons, and the dynamics approach the linear limit.

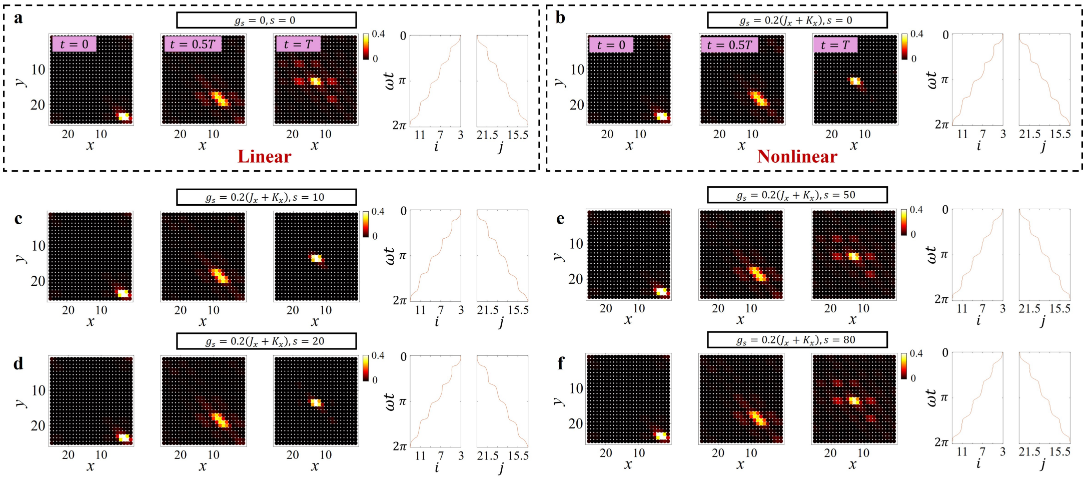


FIG. S21. 2D Thouless pumping behavior dependent on saturation strength under weak nonlinearity. (a) In the linear case, the spatial distributions of the wave packet at three times $t = 0$, $t = 0.5T$, and $t = T$, and the corresponding

c. m. trajectories. (b) In the Kerr-nonlinearity case $(s = 0)$, the spatial distributions and c. m. trajectories of the wave packet for $g_s = 0.2(J_x + K_x)$. (c)-(f) Spatial distributions and c. m. trajectories of the wave packet for $s =$ 10, 20, 50, and 80.

Increasing the nonlinear strength to $g_s = 2.8(J_x + K_x)$, the system exhibits fractional-quantized soliton transport in the Kerr-nonlinearity case ($s = 0$), as shown in Fig. S22a. We then compute the wave packet evolution and the corresponding c. m. trajectories for $s = 0.05$, 1, 30, 120 and 700, as shown in Figs. S22b-S22f. We find that the transport of the wave packet is highly sensitive to the parameter $s$: fractional transport occurs only when $s$ is very small (e.g., $s = 0.1$). When $s$ increases to 1, the wave packet starts to spread, and the fractional transport behavior is gradually lost. However, as $s$ increases to 30, the wave packet begins to propagate in the opposite direction while still expanding outward. When $s$ increases to 120, the wave packet becomes a soliton and exhibits integer-quantized transport. Further increasing $s$ to 700 drives the system from integer-quantized soliton transport to linear transport, after which the behavior stabilizes and no longer changes with increasing $s$.

These results show that increasing the parameter $s$ causes the nonlinearity to saturate at lower intensities, thereby weakening the nonlinear effect. As a result, for moderate nonlinear strength $2.8(J_x + K_x)$, increasing $s$ causes the system to gradually transition from fractional-quantized soliton transport to integer-quantized soliton transport and finally to linear transport.

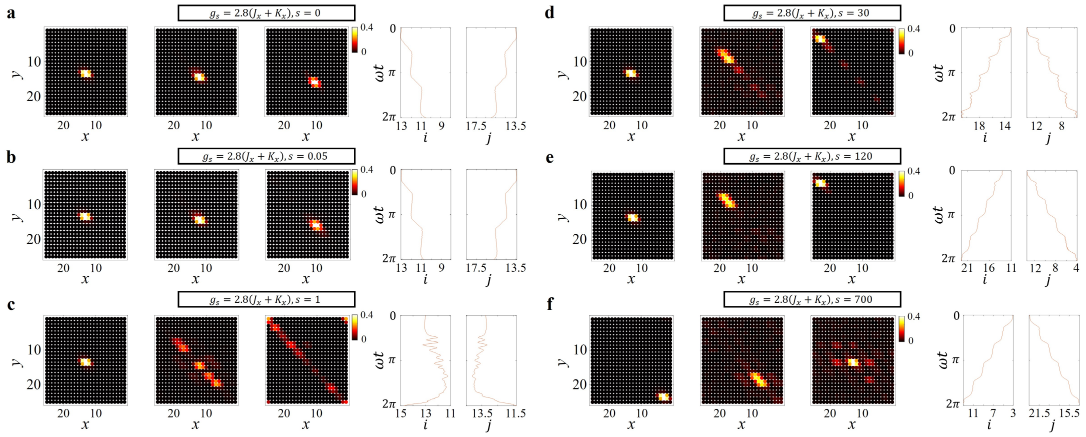


FIG. S22. 2D Thouless pumping behavior dependent on saturation strength under moderate nonlinearity. (a)-(f) The transport behavior of the wave packet and the corresponding c. m. trajectories for the nonlinear strength fixed at $g_s = 2.8(J_x + K_x)$ and parameter $s$ at 0, 0.05, 1, 30, 120, and 700.

Finally, for a stronger nonlinearity $g_s = 5(J_x + K_x)$, varying $s$ allows us to realize four distinct

types of quantized transport, see Figs. S23a-S23e. In this strong-nonlinearity regime, at $s = 0$ (Kerr) the system shows trapped soliton transport. Increasing $s$ to a small value ($s = 0.1$) keeps the soliton trapped. As $s$ is further increased to 0.7, 150, and 900, the system evolves from trapped to fractional soliton transport, then to integer soliton transport, and eventually to linear transport.

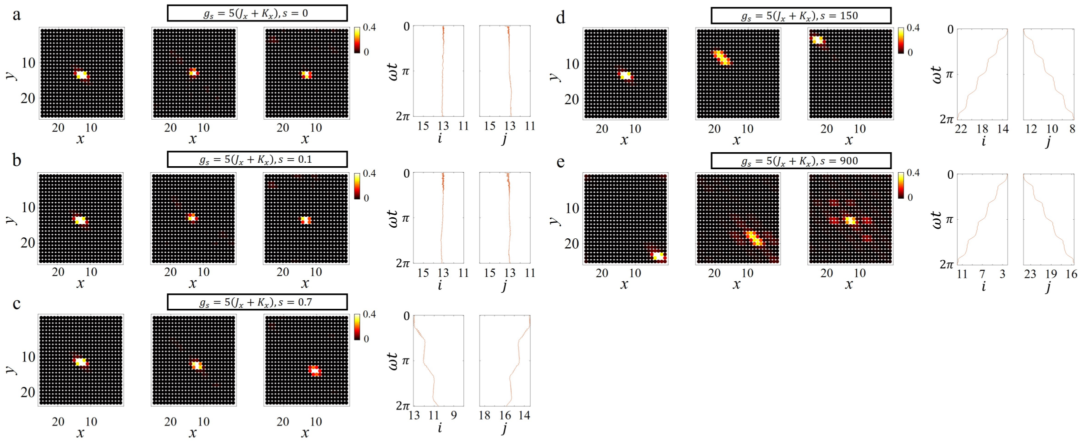


FIG. S23. 2D Thouless pumping behavior dependent on saturation strength under strong nonlinearity. (a)-(e) The transport behavior of the wave packet and the corresponding c. m. trajectories for the nonlinear strength fixed at $g_s = 5(J_x + K_x)$ and parameter $s$ at 0, 0.1, 0.7, 150, and 900.

These results indicate that, after replacing the Kerr nonlinearity with a saturable nonlinearity, integer, fractional, and trapped transport behaviors still exist under specific conditions. The difference is that, when the nonlinear strength is fixed at $g_s = 0.2(J_x + K_x)$, $2.8(J_x + K_x)$, and $5(J_x + K_x)$ the quantized transport of the wave packet can be controlled by adjusting the parameter $s$.

## XIX. SAMPLE FABRICATIONS AND CIRCUIT MEASUREMENTS

In this section, we provide a detailed description of the design and fabrication process of the printed circuit boards (PCB). We exploit electric circuits by using LCEDA program software, where the PCB composition, stack-up layout, internal layer and grounding design are suitably engineered. To experimentally observe the nonlinear Thouless pumping phenomenon, three types of printed circuit boards ($PCBs$) are required: $PCB_{IS}$, $PCB_{gain}$, and $PCB_{test}$, each with a quantity of one. Specifically, the $PCB_{IS}$ is used to set the initial voltages matching the eigen-solution of transient solitons. This circuit board incorporates 50 single-pole double-throw switches (ADG1419BCPZ-REEL7), with each module's input connected to an external power supply at different voltage levels. The output

terminals are connected to the corresponding nodes of the test circuit board ($PCB_{test}$). All initial voltage values are consistent with the instantaneous soliton eigen-solutions serving as the initial conditions.

The $PCB_{gain}$ is employed to amplify voltages from an arbitrary waveform generator. It consists of five independent non-inverting amplifiers, each capable of amplifying the input signal by a factor of ten, thereby achieving the required driving voltage amplitude $V_0$.

The $PCB_{test}$ is the nonlinear pump circuit board under examination, comprising three main modules: conventional INICs, time-varying INICs, and third-order nonlinear INICs. The test board features an ample number of probe pins for connecting to the $PCB_{IS}$ and measuring node voltage signals. All capacitors and resistors are in 0603 surface-mount packages. Furthermore, to ensure component tolerance, we used a WK6500B impedance analyzer to select high-precision circuit elements (with a deviation of only 0.1%). Additionally, 0.1uF decoupling capacitors are placed on power supply pins to filter out AC noise and high-frequency interference, ensuring stable DC operating voltages.

In terms of printed circuit board design, the PCB is six-layered. Apart from the top and bottom layers, there are dedicated power supply layers for ±15 V, a signal layers, and an internal ground plane to minimize electric field interference. All internal layers are interconnected via blind and buried vias.

During measurements, operational amplifiers (LT1363) and multipliers (AD633JNZ) are simultaneously powered by an external ±15 V supply. The single-pole double-throw switches (ADG1419BCPZ-REEL7) are driven by an external ±12 V power source. An arbitrary waveform generator (DG70004) and a signal generator (FY2300−12M) output five sinusoidal driving signals at frequencies of 11.49 Hz and 119 Hz, corresponding to the results in Figures 3c and 3d, respectively. The phases are set to 306°, 90°, 234°, 378°, and 162°. After connecting all boards, a step signal is applied to the $PCB_{IS}$, causing all switches to transition from closed to open, thereby assigning an initial voltage to each circuit node on the test board $PCB_{test}$. The moment the switches open, the voltage signals at all sub-nodes begin to evolve freely. Finally, a four-channel oscilloscope (DSO7104B) is used to monitor the voltage signals—three channels are connected to the sub-nodes for signal acquisition, while the remaining channel is linked to the marker signal of the arbitrary waveform generator, with its falling edge serving as the trigger condition.

## REFERENCES

[1]. Lohse M, Schweizer C, Price HM *et al.* Exploring 4D quantum Hall physics with a 2D topological

charge pump. *Nature* 2018; **553**: 55-58.

[2]. Jürgensen M, Mukherjee S, Jörg C et al. Quantized fractional Thouless pumping of solitons. Nat Phys 2023; 19: 420-426.
